# Continuity of Psychopathology Throughout Adolescence and Young Adulthood

**DOI:** 10.1080/15374416.2022.2042695

**Published:** 2022-03-08

**Authors:** Jennifer S. Richards, Catharina A. Hartman, Johan Ormel, Albertine J. Oldehinkel

**Affiliations:** University of Groningen, University Medical Center Groningen, Department of Psychiatry, Interdisciplinary Center Psychopathology and Emotion Regulation

## Abstract

**Objective:**

This study tested two opposing hypotheses on the continuity of psychopathology throughout adolescence and young adulthood; differentiation versus dynamic mutualism. Differentiation predicts that co-occurrence decreases, while dynamic mutualism predicts that co-occurrence increases due to causal interactions amongst mental health problems.

**Method:**

Using data from the Dutch TRacking Adolescents’ Individual Lives Survey (n = 2228, 51% female), we studied the development of self-reported internalizing, externalizing, and attention problems at ages 11 to 26 across six waves. Random-intercept cross-lagged panel modeling was employed to distinguish within-person development from stable between-person processes.

**Results:**

Large stable between-person associations indicated that adolescents with internalizing problems tended to have both externalizing and attention problems as well. On a within-person level, mental health problems showed partial stability and strong cross-sectional co-occurrence. Within-wave associations of internalizing with externalizing or attention problems decreased between age 11 and 16 years, after which they increased again. Little heterotypic continuity was found: age 11 externalizing predicted age 13 attention, which in turn predicted age 16 externalizing problems, and internalizing predicted externalizing problems across ages 22 to 26. Findings were similar for males and females.

**Conclusions:**

Our findings suggest co-occurrence decreases during early and middle adolescence, supporting differentiation. While co-occurrence increased again into young adulthood, this could not be labeled as dynamic mutualism because little evidence for heterotypic continuity was found in this phase of life. The strong stable links between internalizing, externalizing, and attention problems stress the importance of targeting these mental health problems and their shared risk factors together.

Mental health problems often co-occur; it is rather the rule than exception that problems of one disorder are accompanied by problems from a different, presumedly distinct, disorder (Caspi et al., 2020; Kessler et al., 2005). The extent of such co-occurrence may change over the life course. Especially childhood and adolescence are characterized by a waxing and waning of behaviors and symptoms, with the remission of certain disorders, while others arise. The continuity of mental health problems across development can be described in terms of *homotypic* and *heterotypic continuity*. Homotypic continuity refers to the amount of stability mental health problems show over time (e.g., childhood anxiety predicting future anxiety). Heterotypic continuity refers to mental health problems from one disorder predicting problems from another disorder at a later time (e.g., childhood anxiety predicting future aggression; Angold et al., 1999; Rutter et al., 2006). Despite years of research, the mechanisms of how mental health problems codevelop or why heterotypic continuity occurs are not yet clearly understood. Gaining more insight into the underlying processes of developmental co-occurrence is important for effective prevention and intervention strategies. The current study focused on two possible, but divergent, developmental processes that have been proposed to describe the development of psychopathology and its underlying etiology: *differentiation* versus *dynamic mutualism*. Specifically, we studied whether these competing hypotheses explain the continuity of common mental health problems, i.e., internalizing, externalizing, and attention problems, across adolescence into young adulthood.

The differentiation hypothesis suggests that psychopathology becomes increasingly distinct across development, moving from an undifferentiated to a differentiated state. Mental health problems are hypothesized to strongly co-occur at first, after which co-occurrence – and heterotypic continuity – decrease over time as mental health problems become more specific (Murray et al., 2016; Sterba et al., 2010). An example of differentiation would be when a child starts out with multiple co-occurring internalizing and externalizing problems, which later crystalize into aggression problems only. Differentiation is in line with the observation that disorders such as anxiety and depression are more difficult to distinguish in childhood than in adulthood (Lahey et al., 2004). In this theory, co-occurrence is thought to be the result of a general liability or common cause of psychopathology that manifests into specific symptom patterns (Murray et al., 2016; Sterba et al., 2010). In contrast, a network perspective of psychopathology considers co-occurrence to arise from many (direct and indirect) interactions between different mental health problems that reinforce and strengthen each other, leading to increased heterotypic continuity and co-occurrence over time, which is referred to as dynamic mutualism (Cramer et al., 2010; Murray et al., 2016; Van der Maas et al., 2006). Dynamic mutualism corresponds with developmental pathways described by cascade models (Masten & Cicchetti, 2010), in which one symptom or set of symptoms directly causes another across different domains of psychopathology. Unlike differentiation, mutualism does not hypothesize a general liability or common cause underlying co-occurrence, although external factors, such as negative life experiences (Cramer et al., 2010) may affect how mental health problems impact one another as well (e.g., negative life events may reinforce previous attention problems or cause co-occurring depressive problems).

While there are several approaches to study heterotypic continuity (see, e.g., Caspi et al., 2020; Copeland et al., 2013; Lahey et al., 2014; McElroy et al., 2018; Patalay et al., 2017), not all of these are suited to test whether differentiation or dynamic mutualism occurs in development. The latter require examining heterotypic continuity while controlling for concurrent associations and homotypic continuity (Lahey et al., 2014). This allows one to verify that longitudinal links and changes in co-occurrence over time are not confounded by prior co-occurrence. In addition, taking a dimensional approach to studying heterotypic continuity (i.e., focusing on mental health problems instead of categorical disorders) is essential for testing mutualism, as in this theory predictions are specifically made about interactions amongst mental health problems and not disorders. Cross-lagged panel modeling (CLPM) has been a popular choice to study the bidirectional developmental relations among mental health problems (e.g., van der Ende et al., 2016; McElroy et al., 2018; Obsuth et al., 2020; Shevlin et al., 2017; Van Lier et al., 2012). However, the approach has been criticized for not distinguishing within-person from stable between-person processes, hence possibly yielding biased estimates (Berry & Willoughby, 2017; Hamaker et al., 2015). While stable between-person differences provide information about how individuals differ from one another in terms of trait levels, the within-person development describes how changes in psychopathology predict future changes within individuals. As mental health development occurs within persons, it is important to study within-person processes separate from stable between-person differences (Duncan-Jones et al., 1990).

Statistical approaches separating within-person development from between-person processes (such as the random-intercept cross-lagged model, see, Hamaker et al., 2015) are rapidly gaining popularity in developmental research. Still, relatively few studies have investigated the heterotypic continuity of internalizing, externalizing, and attentions problems on a within-person level, while controlling for stable between-person differences. Most examined the developmental relations between internalizing and externalizing problems (Murray et al., 2019; Flouri et al., 2019; Oh et al., 2020), with only two studies including attention problems (Allegrini et al., 2021; Murray et al., 2022). All focused on childhood and/or adolescence (ages 3 to 17 years), using either teacher or parent reports. These studies yielded inconsistent results with respect to the strength and direction of heterotypic development.

Studying heterotypic development assessed annually in children across ages 5 to 9 years, Oh et al. (2020) found that higher levels of externalizing problems stably predicted later higher levels of internalizing problems across all ages, although the reported effect sizes were small (<.10). In contrast, within-person concurrent correlations appeared to increase with age. Similarly, Flouri et al. (2019) found externalizing problems predicted future internalizing problems in children across ages 3, 5, 7, 11 and 14 years. These effects appeared to initially increase followed by a decrease from age 7 for girls and age 11 for boys. In addition, for girls, small effects were found from internalizing to externalizing problems (≦.10) in early childhood (ages 3 to 7 years). Estimates for concurrent associations were not provided. Murray et al. (2019) reported relatively small effects from externalizing to later internalizing problems from ages 7 to 15 years across eight waves. These seemed to diminish across age, with a negative effect from 13 to 15 years, but this was not statistically tested. Further, they found age 12 internalizing negatively predicted age 13 externalizing problems. Here too, no concurrent associations were given. Using data from the same study, Murray et al. (2022) examined heterotypic continuity between anxiety and attention problems at ages 13, 15, and 17 years. Attention problems predicted subsequent anxiety problems, with a smaller effect from ages 15 to 17. In addition, anxiety predicted later attention problems only across ages 15 to 17. In contrast, the concurrent correlations appeared to become stronger over time.

To date, only one study investigated internalizing, externalizing, and attention problems simultaneously, including social problems as well and using two different cohorts of children across mean ages 7, 9 and 12 years (Allegrini et al., 2021). Similar to the findings above, in the first cohort externalizing problems predicted internalizing problems, which in turn predicted later externalizing problems. In addition, they found reciprocal effects between externalizing and attention problems which appeared slightly larger at older ages. Internalizing problems predicted later attention problems across ages 7 to 9 only. However, only the effects from externalizing to later attention and internalizing problems across ages 7 to 9 were replicated in the second cohort. As for the concurrent associations, these seemed to increase across time for the first cohort, but appeared more stable for the second cohort.

Overall, these findings cannot easily be interpreted as either differentiation or mutualism. Looking at the longitudinal predictions, on the one hand, the increasing heterotypic continuity found in early childhood by Flouri et al. (2019) is most in line with mutualism. On the other hand, the decreasing (including negative) effects found in childhood and adolescence (Flouri et al., 2019; Murray et al., 2019; Murray et al., 2022) are more consistent with differentiation. Similarly, the study by Allegrini et al. (2021) found most robust evidence for developmental links at younger ages only, again suggesting differentiation. Examining the change in concurrent associations further complicates the matter. That is, most studies reported an increase in co-occurrence, which was opposite to the developmental changes found in longitudinal heterotypic effects (i.e., stable or decreasing effects). Note that while increasing concurrent associations suggest that relations amongst mental health problems become stronger, when such effects are not accompanied by a pattern of longitudinal heterotypic effects becoming stronger over time, they do not support mutualism. Altogether, there seems to be some support that mutualism may occur in early childhood, followed by a process of differentiation throughout childhood and early adolescence, although the evidence is not overwhelming.

The mixed findings may in part be explained by methodological differences between these studies, such as the type of informant or questionnaire, and the consideration of attention problems. For example, studies focusing only on internalizing and externalizing problems may yield different results than those including attention problems if heterotypic continuity from internalizing to externalizing problems or vice versa runs through attention problems. In addition, it is likely that the small or contrasting heterotypic effects are in part age-related, as especially internalizing problems are more prevalent in adolescence compared to childhood. Considering the major biological, cognitive, and socio-emotional developmental changes that occur during adolescence, this period is a likely candidate for a process of differentiation or dynamic mutualism to unfold. A comparison of the within-person heterotypic associations between internalizing, externalizing, and attention problems from adolescence to young adulthood, therefore, seems timely.

In the current study, we aimed to build upon and extend prior findings on the longitudinal continuity of psychopathology using data from a prospective cohort study of Dutch adolescents spanning the whole period between early adolescence and young adulthood (ages 11 to 26 years).^1^^1^Please note that TRAILS data have previously been used to investigate comorbidity development, but only at younger ages (e.g., Jaspers et al., 2012; Ormel et al., 2015; Roy et al., 2014), and not with regard to internalizing, externalizing and attention problems, while distinguishing within- from between-person levels. We tested whether the development of self-reported internalizing, externalizing, and attention problems within individuals during these formative years can be described as differentiation or dynamic mutualism by examining the homotypic and heterotypic continuity on a within-person level separate from stable between-person differences. If the development of psychopathology is best understood as a process of differentiation, we would expect to find that within individuals, associations between different types of problems, such as internalizing and externalizing problems, become weaker as adolescents become older. That is, both concurrent and longitudinal heterotypic associations are expected to decrease over time (or even become absent or negative). Alternatively, if dynamic mutualism occurs, we would expect to find associations between different types of problems to become stronger over time, as evidenced by an increase in concurrent and longitudinal heterotypic associations. As both models specifically describe the co-development of mental health problems within individuals, we did not have specific hypotheses regarding the stable between-person associations. Finally, because the development of mental health problems during adolescence and young adulthood has been shown to diverge between males and females, we also investigated whether the developmental continuity showed sex differences.

## Method

### Participants

Participants were selected from the TRacking Adolescents’ Individual Lives Survey (TRAILS), which involves bi- or triennial follow-up measurements. Data were used from the first six assessment waves (T1: 2001 - 2002; T6: 2016 - 2017), which took place at mean ages 11.1 (*SD *= .56), 13.6 (*SD* = .61), 16.3 (*SD* = .71), 19.1 (*SD* = .60), 22.3 (*SD* = .65) and 25.7 (*SD* = .60) years, spanning the period from early adolescence until young adulthood. Detailed descriptions of TRAILS, including recruitment and assessment procedures, can be found in previous reports (Huisman et al., 2008; Oldehinkel et al., 2015; Ormel et al., 2012). Children born between October 1989 and September 1991 were recruited via schools from five municipalities in the north of The Netherlands, including both urban and rural areas. Primary school was a prerequisite for inclusion. A total of 135 schools was contacted, of which 13 refused to participate, thereby excluding 338 children. Subsequently, parents of eligible children were informed about the study and invited to participate. After excluding 210 children due to serious health or language problems, 2935 children were invited to participate, of whom 2230 children and their parents enrolled at T1 (response rate 76%, 51% female). Most participants were of Dutch ethnicity (86.5%), and had an average socioeconomic status based on parental education, occupation, and income (25.3% low, 49.5% medium, 25.2% high). The retention throughout the six waves was 73–96%. Extra efforts to retain participants included additional telephone calls, house visits, flexibility in measurement and timing, and a lottery with attractive prizes. Participants who missed one or more follow-up waves during T1 – T5 were more likely to be male, come from low socioeconomic families, and to have more parent-reported externalizing problems at baseline (Oldehinkel et al., 2015). The present study included 2228 participants who had at least one assessment during T1 to T6. An overview of the participant characteristics is shown in Table 1, Table 2.

**Table 1.** Participant characteristics and self-reported problem scores across all waves.

		Female	Age		Internalizing problems		Externalizing problems		Attention problems	
Wave	*N*	*%*	*M*	*SD*	*M*	*SD*	*M*	*SD*	*M*	*SD*
T1	2202	51	11.11	.56	.36	.24	.30	.21	.48	.31
T2	2093	51	13.57	.61	.34	.25	.30	.21	.56	.35
T3	1661	53	16.28	.71	.32	.24	.32	.22	.61	.36
T4	1698	55	19.08	.60	.26	.24	.20	.19	.50	.36
T5	1502	56	22.29	.65	.28	.26	.17	.15	.47	.35
T6	1316	59	25.66	.60	.34	.29	.16	.15	.49	.36

**Table 2.** Pairwise correlations between self-reported problems across all waves.

	Problem score	1	2	3	4	5	6	7	8	9	10	11	12	13	14	15	16	17	18
1	Internalizing T1	-																	
2	Internalizing T2	.50	-																
3	Internalizing T3	.38	.58	-															
4	Internalizing T4	.32	.45	.59	-														
5	Internalizing T5	.27	.40	.54	.59	-													
6	Internalizing T6	.26	.36	.49	.55	.65	-												
7	Externalizing T1	.54	.21	.13	.16	.10	.10	-											
8	Externalizing T2	.30	.46	.28	.25	.19	.15	.44	-										
9	Externalizing T3	.20	.25	.39	.25	.23	.18	.32	.53	-									
10	Externalizing T4	.20	.24	.31	.52	.29	.32	.30	.43	.55	-								
11	Externalizing T5	.17	.24	.29	.35	.54	.36	.24	.35	.50	.58	-							
12	Externalizing T6	.20	.22	.28	.39	.38	.58	.25	.33	.45	.60	.60	-						
13	Attention T1	.58	.32	.22	.21	.19	.17	.57	.31	.24	.22	.17	.21	-					
14	Attention T2	.31	.53	.32	.28	.23	.19	.33	.59	.38	.31	.29	.25	.43	-				
15	Attention T3	.25	.33	.49	.34	.32	.29	.27	.36	.59	.41	.39	.38	.34	.51	-			
16	Attention T4	.22	.29	.36	.58	.35	.39	.23	.31	.37	.60	.40	.45	.30	.42	.56	-		
17	Attention T5	.19	.28	.32	.37	.56	.41	.21	.25	.31	.38	.57	.43	.28	.37	.49	.58	-	
18	Attention T6	.18	.21	.28	.37	.40	.59	.20	.17	.24	.37	.42	.59	.26	.29	.41	.53	.62	-

All correlations significant at *p* < .001.

### Procedure

Each assessment wave was approved by the Dutch ethical committee (CCMO). Participants were treated in accordance with APA ethical standards and the Declaration of Helsinki, and all measurements were carried out with their adequate understanding and written consent. Adolescents provided written consent from T2 onwards. A parent or guardian provided written parental consent for adolescent participation during the first three waves and written consent to participate at each wave. At T1, a home visit was conducted by well-trained interviewers. Parents or guardians (95.6% mothers) were interviewed about their family composition, child’s developmental history, somatic health, impairments and familial psychopathology. In addition, they completed written questionnaires. At waves T2 – T4, parents completed a questionnaire they received via postal mail. Adolescents completed questionnaires in groups at school, under the supervision of at least one research assistant, during T1 to T3. From T4 onwards, participants and parents completed questionnaires online, unless requested otherwise.

### Measures

#### Psychopathology

Psychopathology was assessed with the Youth Self Report ([YSR]; Achenbach & Rescorla, 2001) at T1 - T3 and the Adult Self Report ([ASR]; Achenbach & Rescorla, 2003) at T4 – T6. Good test-retest reliability and adequate content, criterion-related and construct validity has been demonstrated for both the YSR and ASR in similar non-referred populations (Achenbach & Rescorla, 2003; Achenbach & Rescorla, 2001). Participants scored 112 (YSR) or 102 (ASR) symptoms of psychopathology on a 3-point Likert scale (0 = not true; 1 = somewhat/ sometimes true; 2 = very/ often true). In the current study items from the internalizing, externalizing, and attention problems scales were included. For consistency, we selected items from the ASR that matched the YSR items and scales and vice versa; 14 YSR and 35 ASR items could not be matched and were therefore excluded. The two YSR items “I steal at home” and “I steal from other places than home” were combined such that the highest score was selected to match the ASR item “I steal.” Subsequently, mean scores were calculated based on 28 items for internalizing (α_T1-T6_ = .86 – .91), 22 items for externalizing (α_T1-T6_ = .76 – .82) and 7 items for attention problems (α_T1-T6_ = .62 – .72). An overview of the included items can be found in the supporting information, Table S1. At each wave, the mean scores of these adapted scales correlated highly with the standard YSR and ASR internalizing, externalizing, and attention problem scales (*r* = .82 – .99).

#### Covariates

Socioeconomic status (SES) was measured by parental education, occupation, and family income at T1. Parental education was summarized into five categories. Occupational level was categorized using the International Standard Classification of Occupations (Ganzeboom & Treiman, 1996). Low family income was determined as a monthly net income of less than €1135 per month, which is approximately equal to a welfare payment. Mental healthcare use indicated whether participants ever (versus never) received mental healthcare for emotional or behavioral problems (e.g., by a psychologist or psychiatrist) based on parent (T1 – T4) and/or self-reports (T4 – T6). Medication use indicated whether participants ever (versus never) used antipsychotics, anxiolytics, hypnotics/ sedatives, antidepressants or psychostimulants in the previous year preceding baseline, or in the preceding two years for subsequent waves. Information was provided by parents (T1 – T4) and participants (T4 – T6).

### Statistical Analysis

Homotypic and heterotypic continuity of self-reported internalizing, externalizing, and attention problems across T1 – T6 were examined using random intercept cross-lagged panel modeling (RI-CLPM). The RI-CLPM is a modified version of the traditional CLPM, which includes a random intercept to capture stable between-person differences, thereby allowing the study of within-person development (Hamaker et al., 2015). Initially, we planned to run a multiple indicator RI-CLPM (Hamaker, 2018) in which latent measurement factors of internalizing, externalizing, and attention problems were included, after establishing (partial) longitudinal measurement invariance. However, these attempts resulted in model convergence and misspecification issues. The same was true for adopting a two-step approach, i.e., saving factor scores from an invariance model spanning T1 – T6, and subsequently running the RI-CLPM. Therefore, the RI-CLPMs were run with mean internalizing, externalizing, and attention problem scores instead. Results of the longitudinal measurement invariance testing as well as the alternative RI-CLPM models and resulting errors can be found in the supporting information.

**Figure 1.**
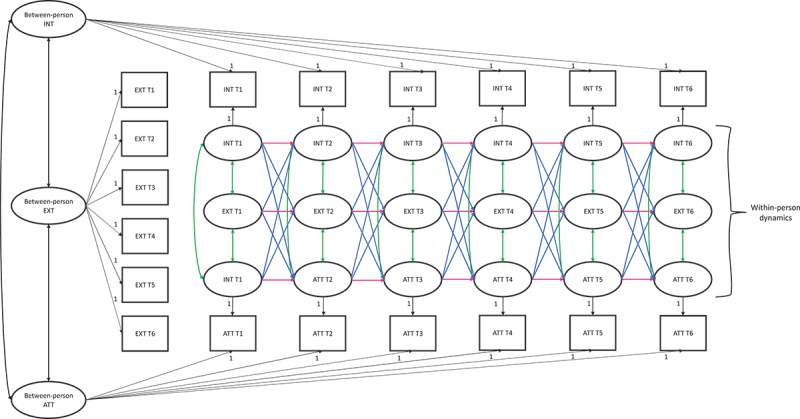
RI-CLPM including three intercepts to capture stable between-person differences and 18 latent variables to estimate within-person changes of internalizing (INT), externalizing (EXT), and attention problems (ATT). Autoregressive paths in pink reflect homotypic continuity; cross-lagged paths in blue reflect heterotypic continuity; correlations at T1 and correlated change at T2 – T6 in green reflect concurrent co-occurrence. For clarity arrows from observed to latent within-person variables of externalizing problems have been omitted.

We first checked whether there was sufficient within-person variance over time by calculating the intraclass correlations (ICCs) for internalizing, externalizing, and attention problems. For each variable, the ICC indicates how much of the total variance across the waves is accounted for by between-person versus within-person variance. A high ICC (>.50) indicates more between-person variance compared to within-person variance, while a low ICC (<.50) indicates proportionally higher within-person variance (Hox, 2002). Next, RI-CLPMs were run by regressing the observed mean scores of internalizing, externalizing, and attention problems on their own latent factor with each loading constrained to 1 for each wave, resulting in 18 within-person latent factors in total, see, Figure 1. Autoregressive (stability) paths, cross-lagged paths, T1 correlation and correlated changes were added between the latent factors of internalizing, externalizing, and attention problems. Three overarching random intercept factors were specified to capture the time-invariant trait-like components of internalizing, externalizing, and attention problems, representing stable differences between individuals (i.e. between-person effects). The observed scores and indicators of these factors, and all factor loadings were constrained to 1. The variances of the observed scores were constrained to zero, thereby capturing all variation in the observed measures by the within- and between-person latent factor structure. The correlations between the overarching latent intercepts reflect how stable between-person differences in internalizing, externalizing and attention problems are related to one another. The within-person dynamics are captured in the stability paths, cross-lagged paths, T1 correlation and correlated changes. The stability paths indicate the extent to which an individuals’ deviation from their own expected problem scores predict future within-person deviations at the next measurement wave, thereby reflecting homotypic continuity. The cross-lagged paths indicate whether deviations from their own problem scores in one domain predict future within-person changes in another domain at the next wave, reflecting heterotypic continuity. The T1 correlations indicate whether individuals’ deviations from their own expected scores in one domain are linked to deviations from their own scores in another, while the within-wave (residual) correlations indicate whether within-person changes in problems scores of one domain are linked to within-person changes in scores of another domain.

Next, we performed a series of difference tests to check whether constraining or freeing estimates across time resulted in a more parsimonious model. First, we tested whether the grand means of internalizing, externalizing, and attention problems were stable across time by constraining the means to be equal across the measurement waves. Second, we checked the assumption that the effects of the random-intercepts are the same at each measurement wave. In the default RI-CLPM the factor loadings of the random intercepts are constrained to 1 at each wave; by freeing these constraints, the factor loadings can be estimated freely and thus vary across time. Third, to test whether path estimates changed over time or not, we compared a fully unconstrained model to models in which stability paths, within-wave associations, and/or cross-lagged paths were constrained to be equal over time in a step-wise manner. Differences in model fit were assessed based on the Satorra-Bentler scaled Chi-square difference tests (Satorra & Bentler, 2001), as well as changes in the Comparative Fit Index (ΔCFI ≥ −0.010), the Root Mean Square Error of Approximation (ΔRMSEA ≥0.015), and the Standardized Root Mean Square Error of Residual (ΔSRMR ≥0.030) following Chen (2007). Model fit was considered good when the model achieved >.90 on CFI, and <.06 on RMSEA, and <.08 on SRMR (Hu & Bentler, 1999). Please note that equality constraints were applied to the unstandardized estimates and not the standardized estimates. As such the standardized coefficients presented in the results section may still differ across time.

All analyses were run in Mplus 7.31 (Muthén & Muthén, 2015) using robust maximum likelihood (MLR) which can handle modest deviations in normality. Missing data was handled with full information maximum likelihood (FIML). FIML uses all available data to estimate a likelihood function for each individual, providing unbiased estimates when data is missing at random (Graham, 2009). Of the included 2228 participants, 1091 (49%) had data on six measurement waves, 348 (16%) had data on five waves, 246 (11%) had data on four waves, 209 (9%) had data on three waves, 241 (11%) had data on two waves, and 93 (4%) had data on one wave. Participants with missing data on one or more follow-up waves (1137 [51%]) scored slightly lower on baseline self-reported internalizing problems compared to those with complete data (*M* = .35, *SD* = .25, versus *M* = .38, *SD =* .24, *p* <.01), but did not differ in baseline externalizing or attention problems. Previous research within 3 and 6-wave RI-CLPMs has shown that a large sample size of >1500 is sufficient to detect small within-person effect sizes ≥ .10, with greater power when more waves are included (Kim, Richards, & Oldehinkel, 2022; Masselink et al., 2018). Therefore, effects of <.10 will not be interpreted. The syntaxes for all final analyses can be found on: www.osf.io/4n9cg.

#### Supplemental Analyses

To investigate possible sex differences, we compared models in which parameters were allowed to be estimated freely for males and females with models in which stability paths, within-wave associations, cross-lagged paths, and between-person associations were constrained to be equal across sex. Sex was specified as a grouping variable and the intercepts of the RI-CLPM latent and observed variables were set to 0 for identification. In addition, constraints from the best fitting RI-CLPM model from the main analyses were applied here as well. Model fit was compared based on the same criteria as described above. When the constrained model had a significantly worse fit than the freely estimated model, parameters were freed following the modification indices until the partially constrained model fit the data as well as the unconstrained model.

#### Sensitivity Analyses

Sensitivity analyses were performed to investigate whether the results were robust when controlling for the effects SES, mental healthcare, and medication use. The covariates were included by regressing the observed scores of internalizing, externalizing, and attention problems on SES, mental healthcare, and medication use (following Mulder & Hamaker, 2020). Following the same approach as in the main analyses, we compared whether constraining or freeing model estimates resulted in a more parsimonious model. Additionally, we checked whether the supplemental analyses on sex differences remained robust while controlling for SES, mental healthcare, and medication use as well.

## Results

### Descriptive Statistics

The participant characteristics and pairwise correlations for internalizing, externalizing, and attention problems from T1 – T6 are shown in Tables 1 and 2, respectively. Within-domain correlations were in the similar range for all problem scores (*r* = .24 – .65), with stronger correlations at later waves. The highest correlations were found between problem scores of two subsequent waves within one domain (e.g., internalizing problems T1 – T2), or between different domains within the same wave (e.g., internalizing and externalizing problems T1), ranging between *r* = .39 – .65. The lowest correlations were found for problems scores between different domains across different waves, ranging between *r* = .10 – .45.

### Continuity of Internalizing, Externalizing, and Attention Problems

The ICC was .44 for internalizing, .38 for externalizing, and .41 for attention problems. This indicates that each variable showed proportionally more within-person than between-person variance.

**Figure 2.**
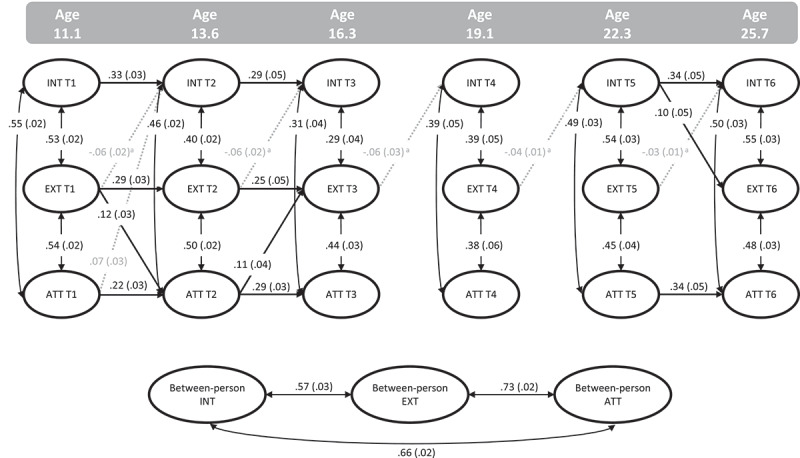
Standardized path estimates (standard errors) from model 16 for internalizing (INT), externalizing (EXT), and attention problems (ATT). ^a^Paths constrained to be equal over time. Dashed gray arrows represent paths <.10, significant at *p* <.05; Solid black arrows represent paths >.10, significant at *p* <.001.

The unconstrained base RI-CLPM model showed good fit: χ^2^ (84) = 351.139; RMSEA = .038; CFI = .981; SRMR = .045. Consecutively checking the constraints on the grand means, random intercepts, stability, within-wave associations, and cross-lagged paths led to a partially constrained model as the best fitting model: χ^2^ (77) = 128.422; RMSEA = .017; CFI = .996; SRMR = .020 (see supplemental Table S4, model 16). In this model the factor loadings of the random intercepts were allowed to vary across time, thereby technically becoming random factors instead of intercepts. In addition, cross-lagged paths from internalizing to attention, and externalizing to internalizing problems were constrained across time. The results are shown in Figure 2 and supplemental Tables S5 and S6.

The effects of the between-person (random) factors varied in strength across time; for all problem scores the factor loadings increased from T1 onwards, with the strongest effects found at T3 and T4, after which effects decreased again, see Table S5. The random factors were strongly associated, indicating relatively strong stable associations between internalizing, externalizing, and attention problems across all waves.

Moderate within-person stability was found for all self-reported problems across T1 – T3. For internalizing and attention problems, moderate stability estimates across T5 – T6 were found as well. For externalizing problems, all stability paths were non-significant after T3. As such, homotypic continuity appeared mostly limited to the first three measurement waves. At each wave there were moderate-to-strong heterotypic associations between within-person changes of internalizing, externalizing, and attention problems. The associations of internalizing with externalizing and attention problems decreased from T1 to T3 (.53 – .29; .55 – .31 respectively), after which they increased from again from T3 to T6 (.39 – .55; .39 – .50 respectively). Such a u-shape pattern was not found between externalizing and attention problems, indicating more stable concurrent associations over time. There was little evidence of longitudinal heterotypic continuity as we found only three significant cross-lagged paths larger than .10. Externalizing problems at T1 predicted within-person changes in attention problems at T2, which in turn predicted within-person changes of externalizing problems at T3. In addition, T5 internalizing problems predicted T6 externalizing problems. Further, some small significant effects were found which fell below <.10: externalizing problems negatively predicted internalizing problems at subsequent waves, and attention problems at T1 predicted internalizing problems at T2.

### Supplemental Analyses

**Figure 3.**
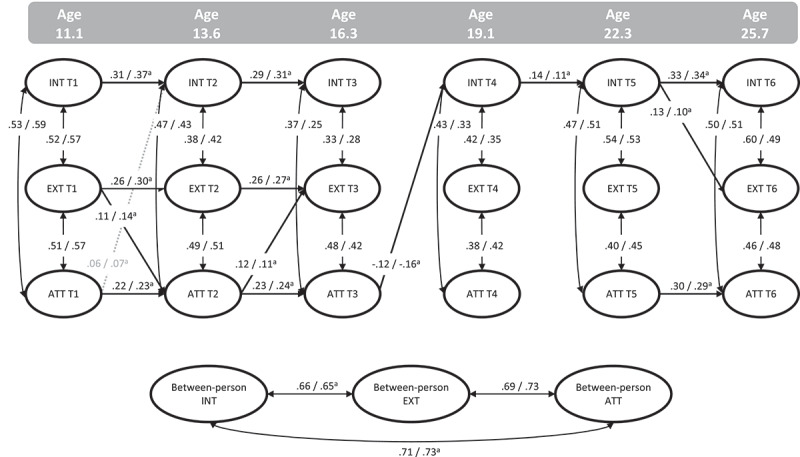
Standardized path estimates for females/males from model 8 for internalizing (INT), externalizing (EXT), and attention problems (ATT). ^a^Paths constrained to be equal for males and females. Dashed gray arrows represent paths < .10, significant at *p* < .05; Solid arrows represent paths >.10, significant at *p* < .001.

The model with equality constraints across males and females (in addition to the time constraints as in the main analysis model 16) showed good model fit (χ^2^(227) = 380.115; RMSEA = .025; CFI = .989; SRMR = .045), albeit significantly worse than the unconstrained model (ΔSBχ^2^ (58) = 145.35, *p* < .001, see supplemental Table S7). Consequently, equality constraints were freed one by one following the modification indices. Following model 5 there were no more recommended modification indices, therefore, within-wave associations across all waves were released step-by-step. Freeing these estimates (model 8) resulted in a model fit which was no longer significantly worse than the unconstrained model: χ^2^ (208) = 280.595; RMSEA = .018; CFI = .995; SRMR = .042; ΔSBχ^2^ (39) = 52.13, *p* = .078. The results of the final partially constrained model are shown in Figure 3 and supplemental Tables S8A and S8B. In addition to the time-constraints, all stability, cross-lagged paths, and the between-person associations of internalizing with externalizing and attention problems were constrained to be equal across males and females.

The estimates for males and females were very similar, and similar to the results of the main analysis. Different from the main analyses, the stability path for internalizing problems across T4 to T5 was significant, as was a negative effect from attention T3 to internalizing problems T4 for both males and females (note that both effects were nominally significant [*p* < .06] in the main analyses). The largest differences between males and females were found for the within-wave associations, where estimates of concurrent correlations were slightly higher for males at T1, but lower at T3. The u-shape pattern of concurrent comorbidity found in the main analysis between internalizing problems with externalizing and attention problems, appeared mostly present for males (T1 to T3: .57 – .28; .59 – .25 and T3 to T6: .28 – .49; .25 – .51, respectively). Females showed smaller changes over time, especially for the associations between internalizing and attention problems (T1 to T3: .52 – .33; .53 – .37 and T3 to T6: .33 – .60; .37 – .50, respectively).

### Sensitivity Analyses

Rerunning the unconstrained base RI-CLPM model while controlling for SES, mental healthcare, and medication use resulted in a good model fit (χ^2^(84) = 318.694; RMSEA = .035; CFI = .984; SRMR = .032). Similar to the main analyses, a partially constrained model in which the factor loadings for the random factors were freed, and the cross-lagged paths from internalizing to attention, externalizing to internalizing, and from externalizing to attention problems were constrained to be equal across time, showed the best model fit (Table S9, Model 17: χ^2^ (81) = 131.488; RMSEA = .017; CFI = .997; SRMR = .017). Overall, including the control variables yielded highly similar results to the models without controls (Table S10). The largest differences were that the between-person associations became slightly attenuated, the small cross-lagged effect from T5 internalizing to T6 externalizing problems became non-significant (*β *= .09, *p* = .057 instead of *β *= .10, *p* = .037 in the main analyses), while the stability effect of internalizing problems from T4 to T5 now was significant (*β *= .20, *p* = .002 instead of *β *= .13, *p* = .058 in the main analyses). In addition, the small (<.10) constrained lagged effects between externalizing and internalizing problems were non-significant (with the exception of the cross-lagged effect from T2 to T3), while the small (<.10) constrained lagged effects between externalizing and attention problems now were significant for all waves.

Next, we reran the final supplemental model on sex differences while including SES, mental healthcare and medication use. This resulted in a good model fit (χ^2^ (266) = 374.832; RMSEA = .019; CFI = .993; SRMR = .043), and again in highly similar results. The only differences were that the between-person associations were slightly smaller, and the lagged effect from T3 attention to T4 internalizing problems became non-significant (see Table S11) for both males and females. As such, the findings of this study appear robust when controlling for SES, mental healthcare, and medication use.

## Discussion

This study examined whether the continuity of common psychopathology over time can be described in terms of differentiation or dynamic mutualism. Specifically, we extended previous literature by investigating the within-person co-development of self-reported internalizing, externalizing, and attention problems across adolescence into young adulthood (ages 11 to 26 years), separate from stable between-person differences. Within-person homotypic continuity was considerable across the first three waves, meaning that here adolescents’ increases of mental health problems – compared to their own typical mental health – predicted future increases of their problems within the same domain. For internalizing and attention problems within-person stability was also found from T5 to T6, suggesting age-dependent homotypic continuity across adolescence and young adulthood. Concurrent co-occurrence was evident as well: at each wave, within-person changes in mental health problems were associated with one another. The strength of associations between internalizing problems and externalizing or attention problems decreased from T1 – T3, and increased again from T3 – T6. This pattern was most apparent for males. In contrast, little heterotypic continuity was found. Overall, within-person changes in problems of one domain did not predict changes in problem-levels of other domains. Exceptions were a significant path from T1 externalizing to T2 attention to T3 externalizing problems, and internalizing predicting externalizing problems across T5 – T6. The strong between-person associations indicated high co-occurrence amongst stable levels of mental health problems across adolescence and young adulthood. Below we discuss the findings in light of differentiation and dynamic mutualism.

Looking at the developmental changes in concurrent links, we found that the co-occurrence of internalizing with externalizing and attention problems initially became weaker (ages 11 to 16) followed by an increase in strength over time (ages 16 to 26). This seems to suggest a process of differentiation in adolescence and dynamic mutualism from late adolescence to young adulthood. Yet, dynamic mutualism explicitly predicts positive interactions amongst mental health problems that become stronger over time (i.e., heterotypic continuity), which we did not find. Thus, while co-occurrence increased, this does not seem to reflect a process of dynamic mutualism. We did find heterotypic continuity between externalizing and attention problems, in that adolescents’ reporting more externalizing problems at age 11 were at risk of more attention problems at age 13, and subsequently at increased risk of externalizing problems at age 16. As we found no further associations at older ages, heterotypic continuity between externalizing and attention problems appeared to diminish across adolescence, suggesting a process of differentiation occurs here as well.

In addition to within-person changes across development, we found that adolescents reporting higher levels of mental health problems in one domain across the study waves, reported higher levels of other problems as well, and vice versa. Such between-person differences may be the result of time-invariant shared risk factors that simultaneously make children vulnerable for the co-development of internalizing, externalizing, and attention problems (Oh et al., 2020). Indeed, parental and individual characteristics, such as maternal prenatal smoking and executive functioning, have been linked to the stable co-occurrence of internalizing and externalizing problems in childhood (Oh et al., 2020). Furthermore, it is well known that internalizing, externalizing, and attention problems have a strong shared genetic background (Cross-Disorder Group of the Psychiatric Genomics Consortium, 2019).

Together, our findings suggest that it may be too simplistic to consider the continuity of psychopathology in terms of only differentiation or dynamic mutualism. While some form of differentiation may occur in adolescence, in young adulthood, neither developmental process seems to apply. Findings from previous studies on within-person associations between internalizing, externalizing, and attention problems (Murray et al., 2019; Allegrini et al., 2021; Flouri et al., 2019; Murray et al., 2022; Oh et al., 2020) are mixed, but provide some tentative support that mutualism may occur in early childhood and differentiation in late childhood and early adolescence. The latter is in line with findings of the current study, and together would suggest an initial process of mutualism, in which comorbidity increases, followed by a period of differentiation throughout adolescence. However, these studies mostly reported within-person effects from externalizing to internalizing problems, which we did not observe. Possibly, cascading interactions between externalizing and internalizing problems occur earlier in development. Methodological differences may play a role here as well. For example, most previous work did not focus on attention problems, and the study of Flouri et al. (2019) included cognitive ability. This is important because including other variables may change the interpretation. For example, by including attention problems, we examined how internalizing problems predicted later externalizing problems while controlling for changes in and links with attention problems. As such, for those studies only including internalizing and externalizing problems, we do not know whether their findings would be the same while including attention problems as well.

In addition, previous studies relied on parent or teacher reports, while we used self-reports. To investigate the potential role of informants, we compared our results of self-reported psychopathology with reports by parents available for T1 – T3 in supplemental analyses (see supporting information). Despite the fact that the self- and parent-report models spanned different periods and hence the stable and within-person variances had different meanings, overall, the findings were similar. A few exceptions were that the estimates of homotypic continuity were weaker, especially for internalizing problems, and there was no pattern of differentiation in concurrent co-occurrence nor a cascading path between externalizing and attention problems. Instead, externalizing problems at age 13 predicted internalizing problems at age 16, an effect not observed in the main analyses. This suggests that the type of informant is relevant when assessing the continuity of psychopathology, as well as whether patterns of differentiation or dynamic mutualism occur. Ultimately, combining reports may provide the most robust information on the development of comorbidity, yet this does not come without challenges. In (early) childhood, self-report is often not feasible or less reliable than other reports of informants, especially for attention and externalizing problems. Over time, children become better at reporting mental health problems and, especially in adolescence and for internalizing problems, parents may become less reliable informants due to adolescents’ decreased self-disclosure. Hence, the validity of each informant’s report is age-dependent.

Comparing developmental co-occurrence between males and females, we found that, overall, both within-person and stable-between person associations amongst internalizing, externalizing, and attention problems were largely similar for males and females across adolescence and young adulthood. The only exception was that the pattern of initially decreased followed by increased co-occurrence was more apparent in males than females: the concurrent co-occurrence between internalizing and externalizing or attention problems did not decrease as strongly from T1 to T3 for females as for males. However, the differences in estimates between males and females were quite small, so we prefer not to speculate about possible reasons for this sex difference. Other studies have not found strong sex differences for the within-person development of internalizing and externalizing problems either (Murray et al., 2019; Flouri et al., 2019), suggesting overall that the continuity of psychopathology is similar rather than different for males and females.

Our study complements previous research employing alternative methods to studying heterotypic continuity. For example, a number of studies have investigated heterotypic continuity by studying changes in disorder or diagnosis (e.g., Caspi et al., 2020; Copeland et al., 2013; Lahey et al., 2014; Shevlin et al., 2017). Overall, such studies have found strong heterotypic continuity in the sense that one type of disorder predicts one or various different disorders at subsequent time points across childhood and adolescence (e.g., Copeland et al., 2013; Shevlin et al., 2017) and into adulthood (e.g., Caspi et al., 2020). In addition to variable-centered approaches, others have focused on person-centered techniques. Such studies have found that mental health problems tend to occur more in some people than others (e.g., Patalay et al., 2017), with some individuals more likely to display homotypic continuity, while for others the development of mental health is characterized by heterotypic continuity. However, while suited for examining the change in mental health over time, these approaches do not allow for testing whether such changes are the result of mental health problems having (bi)directional effects on another, which is relevant for testing dynamic mutualism.

### Clinical Implications

From a clinical perspective, our findings our relevant as well. First, at each assessment wave within-person changes in mental health problems strongly co-occurred across adolescence and young adulthood. The strength of this co-occurrence changed around the age of 16, becoming increasingly stronger hereafter. This is in line with the observation that adolescence is an important transitioning period in terms of mental health. Further, the homotypic continuity found across the first three waves suggests that interventions should target early adolescence to prevent further progression of mental health problems. Our findings also suggest young adulthood is an important period for intervention programs to target further progression of internalizing and attention problems. In addition, we found that internalizing problems predicted later externalizing problems across 22 to 26 years. As such, reducing internalizing problems in young adulthood may be important for preventing later co-occurring externalizing problems as well. Further, although the effects were small, our results highlight a potential causal pathway between externalizing and attention problems in mid-adolescence (11–16 years). Targeting early externalizing problems may help reduce later development of attention problems and subsequent progression of externalizing problems. Further, the strong co-occurrence between stable levels of internalizing, externalizing, and attention problems in adolescence and young adulthood suggest shared stable risk factors underlying comorbidity, which may have exerted their influence on developing co-occurrence in childhood or point to a shared genetic background. Unraveling which shared risk factors are most influential is an important point for future research.

### Strengths and Limitations

Strengths of the current study include the large prospective population cohort spanning adolescence to young adulthood, distinguishing within- from stable between-person associations, and the comparison between males and females. Some limitations should be mentioned as well. First, the time-lag of two to three years between the study waves might be too large to capture all relevant within-person changes in internalizing, externalizing, and attention problems. Similarly, the time-frame of six months for which individuals are asked to report about their mental health problems in the YSR/ ASR may be too long to reflect state-like processes, possible resulting in within-person changes being missed as well. As such, the fairly strong concurrent associations we found may actually reflect heterotypic continuity at a smaller time-frame. Future studies with shorter intervals and state time-frames are needed to clarify whether there is more heterotypic continuity in adolescence and young adulthood when considering a shorter follow-up time and state measurement. That being said, there is a trade-off between the developmental span and the intensity with which psychopathology development can be studied, as increasing the number of measurements increases the participant burden. For example, monthly assessments may not be feasible over a period of ten years. Ultimately, studies varying in intensity and length offer complementary information, and together provide a unified picture of mental health development. Further, we cannot rule out that our findings may (in part) be explained by unmeasured factors, such as social experiences or stressful life events. Another limitation was that psychopathology was measured by the YSR from T1 – T3, and the ASR from T4 – T6. The change in questionnaire reflects normative developmental changes across adolescence (e.g., the YSR-item “afraid to go to school” is not included in the ASR). We tried to minimize the differences by only including matching items across both questionnaires. In addition, we tested for measurement invariance across all waves and found partial invariance across T1 – T6. We had planned to use either a measurement model RI-CLPM or factor scores derived from the invariance testing, but unfortunately both options ran into model fitting difficulties (see supporting information). Therefore, we used mean scores instead, but we cannot guarantee that each problem domain reflects a similar concept for adolescents over time. Next, we were only able to compare informant differences for the first 3 waves. It would be interesting to compare differences for later ages as well, but this was not possible (see discussion above). Finally, participants came from the TRAILS population cohort in the Netherlands, we do not know if the results translate to different population groups with for example, a more extreme socioeconomic status distribution, or other ethnicity groups.

### Conclusions

Our findings suggest that the development of psychopathology in adolescence and young adulthood is characterized by both stable differences between individuals, as well as within-person stability and change. Differentiation was partly supported by the decreases in co-occurrence found from early to mid-adolescence. However, neither differentiation nor dynamic mutualism describe the increased co-occurrence found from late adolescence to young adulthood, as we found little evidence for heterotypic continuity within adolescents. This, together with the strong stable links found between internalizing, externalizing, and attention problems, suggest the relevance of shared risk factors in the development of mental health problems.

## Supplementary Material

Supplemental Material

## Data Availability

For more information on the data supporting the analyses and results, please contact the corresponding author J.S. Richards.

## References

[cit0001] Achenbach, T. M., & Rescorla, L. A. (2001). *Manual for the ASEBA school-age forms & profiles*. University of Vermont.

[cit0002] Achenbach, T. M., & Rescorla, L. A. (2003). *Manual for the ASEBA adult forms & profiles*. University of Vermont.

[cit0003] Allegrini, A., van Beijsterveldt, T., Boomsma, D., Rimfeld, K., Pingault, J.-B., Plomin, R., Bartels, M., & Nivard, M. G. (2021, May 7). Developmental co-occurrence of psychopathology dimensions in childhood: Between and within person processes. 10.31234/osf.io/t486z. PMC10242955 37431387

[cit0004] Angold, A., Costello, E. J., & Erkanli, A. (1999). Comorbidity. *Journal of Child Psychology and Psychiatry*, 40(1), 57–87. 10.1111/1469-7610.00424 10102726

[cit0005] Berry, D., & Willoughby, M. T. (2017). On the practical interpretability of cross‐lagged panel models: Rethinking a developmental workhorse. *Child Development*, 88(4), 1186–1206. 10.1111/cdev.12660 27878996

[cit0006] Caspi, A., Houts, R. M., Ambler, A., Danese, A., Elliott, M. L., Hariri, A., Harrington, H., Hogan, S., Poulton, R., Ramrakha, S., Rasmussen, L. J. H., Reuben, A., Richmond-Rakerd, L., Sugden, K., Wertz, J., Williams, B. S., & Moffitt, T. E. (2020). Longitudinal assessment of mental health disorders and comorbidities across 4 decades among participants in the Dunedin birth cohort study. *JAMA Network Open*, 3(4), e203221. 10.1001/jamanetworkopen.2020.3221 32315069 PMC7175086

[cit0007] Chen, F. F. (2007). Sensitivity of goodness of fit indexes to lack of measurement invariance. *Structural Equation Modeling: A Multidisciplinary Journal*, 14(3), 464–504. 10.1080/10705510701301834

[cit0008] Copeland, W. E., Shanahan, L., Erkanli, A., Costello, E. J., & Angold, A. (2013). Indirect comorbidity in childhood and adolescence. *Frontiers in Psychiatry*, 4, 144. 10.3389/fpsyt.2013.00144 24204349 PMC3816229

[cit0009] Cramer, A. O. J., Waldorp, L. J., van der Maas, H. L. J., & Borsboom, D. (2010). Comorbidity: A network perspective. *Behavioral and Brain Sciences*, 33(2–3), 137–150. 10.1017/S0140525X09991567 20584369

[cit0010] Cross-Disorder Group of the Psychiatric Genomics Consortium. (2019). Genomic relationships, novel loci, and pleiotropic mechanisms across eight psychiatric disorders. *Cell*, 179(7), 1469–1482.e11. 10.1016/j.cell.2019.11.020 31835028 PMC7077032

[cit0011] Duncan-Jones, P., Fergusson, D. M., Ormel, J., & Horwood, L. J. (1990). A model of stability and change in minor psychiatric symptoms: Results from three longitudinal studies. *Psychological Medicine. Monograph Supplement*, 18, 1–28. 10.1017/S0264180100000813 2236383

[cit0012] Flouri, E., Papachristou, E., Midouhas, E., Ploubidis, G. B., Lewis, G., & Joshi, H. (2019). Developmental cascades of internalising symptoms, externalising problems and cognitive ability from early childhood to middle adolescence. *European Psychiatry*, 57, 61–69. 10.1016/j.eurpsy.2018.12.005 30677550

[cit0013] Ganzeboom, H., & Treiman, D. J. (1996). Internationally comparable measures of occupational status for the 1988 international standard classification of occupations. *Social Science Research*, 25(3), 201–239. 10.1006/ssre.1996.0010

[cit0014] Graham, J. W. (2009). Missing data analysis: Making it work in the real world. *Annual Review of Psychology*, 60(1), 549–576. 10.1146/annurev.psych.58.110405.085530 18652544

[cit0015] Hamaker, E. (2018). *How to run a multiple indicator RI-CLPM with Mplus*. http://www.statmodel.com/download/RI-CLPM.pdf

[cit0016] Hamaker, E. L., Kuiper, R. M., & Grasman, R. P. (2015). A critique of the cross-lagged panel model. *Psychological Methods*, 20(1), 102. 10.1037/a0038889 25822208

[cit0017] Hox, J. (2002). *Multilevel analysis: Techniques and applications*. Erlbaum.

[cit0018] Hu, L., & Bentler, P. M. (1999). Cutoff criteria for fit indexes in covariance structure analysis: Conventional criteria versus new alternatives. *Structural Equation Modeling: A Multidisciplinary Journal*, 6(1), 1–55. 10.1080/10705519909540118

[cit0019] Huisman, M., Oldehinkel, A. J., de Winter, A., Minderaa, R. B., de Bildt, A., Huizink, A. C., Verhulst, F. C., & Ormel, J. (2008). Cohort profile: The Dutch ‘TRacking Adolescents’ Individual Lives’ Survey’; TRAILS. *International Journal of Epidemiology*, 37(6), 1227–1235. 10.1093/ije/dym273 18263649

[cit0020] Jaspers, M., de Winter, A. F., Huisman, M., Verhulst, F. C., Ormel, J., Stewart, R. E., & Reijneveld, S. A. (2012). Trajectories of psychosocial problems in adolescents predicted by findings from early well-child assessments. *Journal of Adolescent Health*, 51(5), 475–483. 10.1016/j.jadohealth.2012.02.007 23084169

[cit0021] Kessler, R. C., Chiu, W. T., Demler, O., Merikangas, K. R., & Walters, E. E. (2005). Prevalence, severity, and comorbidity of 12-month DSM-IV disorders in the national comorbidity survey replication. *Archives of General Psychiatry*, 62(6), 617–627. 10.1001/archpsyc.62.6.617 15939839 PMC2847357

[cit0022] Kim, Y., Richards, J. S., & Oldehinkel, A. J. (2022). Self-control, mental health problems, and family functioning in adolescence and young adulthood: Between-person differences and within-person effects *Journal of Youth and Adolescence*. 10.1007/s10964-021-01564-3 PMC9090846 35041145

[cit0023] Lahey, B. B., Applegate, B., Waldman, I. D., Loft, J. D., Hankin, B. L., & Rick, J. (2004). The structure of child and adolescent psychopathology: Generating new hypotheses. *Journal of Abnormal Psychology*, 113(3), 358–385. 10.1037/0021-843X.113.3.358 15311983

[cit0024] Lahey, B. B., Zald, D. H., Hakes, J. K., Krueger, R. F., & Rathouz, P. J. (2014). Patterns of heterotypic continuity associated with the cross-sectional correlational structure of prevalent mental disorders in adults. *JAMA Psychiatry*, 71(9), 989–996. 10.1001/jamapsychiatry.2014.359 24989054 PMC4160409

[cit0025] Masselink, M., Roekel, E. V., Hankin, B. L., Keijsers, L., Lodder, G. M. A., Vanhalst, J., Verhagen, M., Young, J. F., & Oldehinkel, A. J. (2018). The longitudinal association between self‐esteem and depressive symptoms in adolescents: Separating between‐person effects from within‐person effects. *European Journal of Personality*, 32(6), 653–671. 10.1002/per.2179 31105382 PMC6519152

[cit0026] Masten, A. S., & Cicchetti, D. (2010). Developmental cascades. *Development and Psychopathology*, 22(3), 491–495. 10.1017/S0954579410000222 20576173

[cit0027] McElroy, E., Belsky, J., Carragher, N., Fearon, P., & Patalay, P. (2018). Developmental stability of general and specific factors of psychopathology from early childhood to adolescence: Dynamic mutualism or p‐differentiation? *Journal of Child Psychology and Psychiatry*, 59(6), 667–675. 10.1111/jcpp.12849 29197107 PMC6001631

[cit0028] Mulder, J. D., & Hamaker, E. L. (2020). Three extensions of the random intercept cross-lagged panel model. *Structural Equation Modeling: A Multidisciplinary Journal*, 28(4), 1–11. 10.1080/10705511.2020.1784738

[cit0029] Murray, A. L., Caye, A., McKenzie, K., Auyeung, B., Murray, G., Ribeaud, D., Freeston, M., & Eisner, M. (2022). Reciprocal developmental relations between ADHD and anxiety in adolescence: A within-person longitudinal analysis of commonly co-occurring symptoms. *Journal of Attention Disorders*, 26 (1), 109–118. 10.1177/1087054720908333 32172640

[cit0030] Murray, A. L., Eisner, M., & Ribeaud, D. (2016). The development of the general factor of psychopathology ‘p factor’ through childhood and adolescence. *Journal of Abnormal Child Psychology*, 44(8), 1573–1586. 10.1007/s10802-016-0132-1 26846993

[cit0031] Murray, A. L., Eisner, M., & Ribeaud, D. (2019). Within‐person analysis of developmental cascades between externalising and internalising problems. *Journal of Child Psychology and Psychiatry*, 61(6), 681–688. 10.1111/jcpp.13150 31674664

[cit0032] Muthén, L., & Muthén, B. (2015). *Mplus user’s guide* (7th ed.). Muthén & Muthén.

[cit0033] Obsuth, I., Murray, A. L., Folco, S. D., Ribeaud, D., & Eisner, M. (2020). Patterns of homotypic and heterotypic continuity between ADHD symptoms, externalising and internalising problems from age 7 to 15. *Journal of Abnormal Child Psychology*, 48 (2), 1–14. 10.1007/s10802-019-00592-9 31705348 PMC6969859

[cit0034] Oh, Y., Greenberg, M. T., and Willoughby, M. T., &Family Life Project Key Investigators. (2020). Examining longitudinal associations between externalizing and internalizing behavior problems at within- and between-child levels. *Journal of Abnormal Child Psychology*, 48 (4), 1–14. 10.1007/s10802-019-00614-6 31925637 PMC8233408

[cit0035] Oldehinkel, A. J., Rosmalen, J. G., Buitelaar, J. K., Hoek, H. W., Ormel, J., Raven, D., Reijneveld, S. A., Veenstra, R., Verhulst, F. C., Vollebergh, W. A., & Hartman, C. A. (2015). Cohort profile update: The TRacking Adolescents’ Individual Lives Survey (TRAILS). *International Journal of Epidemiology*, 44(1), 76–76n. 10.1093/ije/dyu225 25431468 PMC4339762

[cit0036] Ormel, J., Oldehinkel, A. J., Sijtsema, J., van Oort, F., Raven, D., Veenstra, R., Vollebergh, W. A., & Verhulst, F. C. (2012). The TRacking Adolescents‘ Individual Lives Survey (TRAILS): Design, current status, and selected findings. *Journal of the American Academy of Child and Adolescent Psychiatry*, 51(10), 1020–1036. 10.1016/j.jaac.2012.08.004 23021478

[cit0037] Ormel, J., Raven, D., van Oort, F., Hartman, C. A., Reijneveld, S., Veenstra, R., Vollebergh, W., Buitelaar, J., Verhulst, F. C., & Oldehinkel, A. J. (2015). Mental health in Dutch adolescents: a TRAILS report on prevalence, severity, age of onset, continuity and co-morbidity of DSM disorders. *Psychological Medicine*, 45(2), 345–360. 10.1017/S0033291714001469 25066533

[cit0038] Patalay, P., Moulton, V., Goodman, A., & Ploubidis, G. B. (2017). Cross-domain symptom development typologies and their antecedents: Results from the UK millennium cohort study. *Journal of the American Academy of Child and Adolescent Psychiatry*, 56(9), 765–776.e2. 10.1016/j.jaac.2017.06.009 28838581

[cit0039] Roy, A., Oldehinkel, A. J., Verhulst, F. C., Ormel, J., & Hartman, C. A. (2014) Anxiety and disruptive behavior mediate pathways from attention-deficit/hyperactivity disorder to depression. *Journal of Clinical Psychiatry*, 75(2), e108–13. 10.4088/jcp.13m08648 24602257

[cit0040] Rutter, M., Kim-Cohen, J., & Maughan, B. (2006). Continuities and discontinuities in psychopathology between childhood and adult life. *Journal of Child Psychology and Psychiatry*, 47(3–4), 276–295. 10.1111/j.1469-7610.2006.01614.x 16492260

[cit0041] Satorra, A., & Bentler, P. M. (2001). A scaled difference chi-square test statistic for moment structure analysis. *Psychometrika*, 66(4), 507–514. 10.1007/BF02296192 PMC2905175 20640194

[cit0042] Shevlin, M., McElroy, E., & Murphy, J. (2017). Homotypic and heterotypic psychopathological continuity: A child cohort study. *Social Psychiatry and Psychiatric Epidemiology*, 52(9), 1135–1145. 10.1007/s00127-017-1396-7 28550520 PMC5581823

[cit0043] Sterba, S. K., Copeland, W., Egger, H. L., Costello, J. E., Erkanli, A., & Angold, A. (2010). Longitudinal dimensionality of adolescent psychopathology: Testing the differentiation hypothesis. *Journal of Child Psychology and Psychiatry*, 51(8), 871–884. 10.1111/j.1469-7610.2010.02234.x 20345843 PMC3630513

[cit0044] van der Ende, J. V., Verhulst, F. C., & Tiemeier, H. (2016). The bidirectional pathways between internalizing and externalizing problems and academic performance from 6 to 18 years. *Development and Psychopathology*, 28(3), 855–867. 10.1017/S0954579416000353 27427810

[cit0045] van der Maas, H. L. J., Dolan, C. V., Grasman, R. P. P. P., Wicherts, J. M., Huizenga, H. M., & Raijmakers, M. E. J. (2006). A dynamical model of general intelligence: The positive manifold of intelligence by mutualism. *Psychological Review*, 113(4), 842–861. 10.1037/0033-295x.113.4.842 17014305

[cit0046] van Lier, P. A. C., Vitaro, F., Barker, E. D., Brendgen, M., Tremblay, R. E., & Boivin, M. (2012). Peer victimization, poor academic achievement, and the link between childhood externalizing and internalizing problems. *Child Development*, 83(5), 1775–1788. 10.1111/j.1467-8624.2012.01802.x 22716904

