# Supplementary material for: Continuity of Psychopathology Throughout Adolescence and Young Adulthood
Source: J Clin Child Adolesc Psychol. 2022 Mar 8;53(4):623–36. doi: 10.1080/15374416.2022.2042695 (PMC11318507; doi:10.1080/15374416.2022.2042695)
Supplement: Supplemental Material [file HCAP_A_2042695_SM1674.docx]

**Supporting Information - Continuity of Psychopathology Throughout Adolescence and Young Adulthood**

1. **Longitudinal measurement invariance**

Table S1: Overview included items and parcel distribution for internalizing, externalizing, and attention problems

Table S2: Longitudinal measurement invariance for self-reported internalizing, externalizing, and attention problems T1 - T6

1. **Overview attempted multiple indicator and factor score RI-CLPM models**

Table S3: Overview specified multiple indicator and factor score RI-CLPM models with resulting errors

1. **Self-reported problems**

Table S4: Model fit comparisons for RI-CLPMs with self-reported problems

Table S5: Factor loadings random between-person factors self-reported problems

Table S6: Results from the final constrained RI-CLPM with self-reported problems

1. **Sex differences**

Table S7: Model fit comparisons for RI-CLPMs with self-reported problems of males versus females

Table S8A: Results from final constrained RI-CLPM with self-reported problems for males

Table S8B: Results from final constrained RI-CLPM with self-reported problems for females

1. **Sensitivity analyses**

Table S9: Model fit comparisons for RI-CLPMs with self-reported problems controlling for SES, mental healthcare, and medication use

Table S10: Results from the final constrained RI-CLPM with self-reported problems controlling for SES, mental healthcare, and medication use

Table S11A: Results from the final constrained RI-CLPM with self-reported problems for males controlling for SES, mental health, and medication use

Table S11B: Results from the final constrained RI-CLPM with self-reported problems for females controlling for SES, mental health, and medication use

1. **Parent-reported problems**

Table S12: Model fit comparisons for RI-CLPMs with parent-reported problems

Table S13: Results from final constrained RI-CLPM with parent-reported problems

Figure S1: Path estimates from final constrained RI-CLPM with parent-reported problems

Table S14: Results from the final constrained RI-CLPM with parent-reported problems controlling for SES. Mental healthcare, and medication use

**1. Longitudinal measurement invariance**

**Procedure**

To test the longitudinal measurement invariance across T1 to T6, a series of increasingly constrained confirmatory factor analysis (CFA) models was run simultaneously including internalizing, externalizing, and attention problems. Items for each construct were divided into three so-called parcels (Hau & Marsh, 2004) for which the mean score was then calculated to facilitate computation by reducing model complexity. Model fit was considered good when the model achieved >.90 on the comparative fit index (CFI), <.06 on the root mean square error of approximation (RMSEA), and <.08 on the standardized root mean square residual (SRMR) (Hu & Bentler, 1999). Differences in model fit were assessed based on the change-in-fit indices as recommended by Chen (2007): ΔCFI ≥ −0.010, ΔRMSEA ≥0.015, and ΔSRMR ≥0.030 for metric invariance, and ΔSRMR ≥0.010 for scalar invariance, because SRMR is more sensitive to noninvariance for item loadings than intercepts.

Configural invariance was tested by running the CFA’s across T1 - T6 without any constraints for factor loadings or intercepts. For model identification, all factor variances were fixed to 1, and mean scores fixed to 0. Next, to test for metric invariance, CFA’s were run with factor loadings constrained to be equal across all waves. Factor variances were fixed to 1 at T1 and all means fix to 0. Scalar invariance was tested by constraining both factor loadings and intercepts across all waves. When the scalar model had a significantly worse fit than the freely estimated model, up to 20% of the intercepts were freed following the modification indices until the partially constrained model fit the data as well as the unconstrained model, indicating partial scalar invariance.

**Results and Conclusions**

The results from the longitudinal measurement invariance assessment are shown in Table S2. Both the configural and metric models had an excellent fit to the data, and the metric model did not result in a significantly worse fit than the configural model. Imposing the constraints for scalar invariance, however, did result in a significantly worse fit to the data compared to the metric model. Intercepts were subsequently freed one-by-one until partial scalar invariance was achieved. This ultimately led to model 4e, in which the intercepts for parcel 1 of externalizing problems were freed at T1 and T6, the intercept for parcel 1 of attention problems was freed at T3, and the intercepts for parcel 2 of internalizing problems was freed at T5 and T6. The intercept for the freed parcel of externalizing problems was higher at T1 (.343) and lower at T6 (.239), compared to the constrained intercepts (.280). For attention problems, the freed intercept at T3 was lower (.517 versus .616), and for internalizing problems both freed intercepts at T5 and T6 were slightly higher (.418 and .399 respectively) compared to the constrained intercepts (.371). The results suggest that, overall, the items were interpreted similarly across all waves.

**Table S1.** Overview included items and parcel distribution for internalizing, externalizing, and attention problems

| Internalizing problems | | | | | |
| --- | --- | --- | --- | --- | --- |
| *Parcel 1* | | *Parcel 2* | | *Parcel 3* | |
| 29 | Fears | 5 | Enjoys little | 14 | Cries a lot |
| 33 | Feels unloved | 31 | Fears impulses | 50 | Fearful, anxious |
| 56A | Aches | 35 | Feels worthless | 51 | Dizziness |
| 56C | Nausea | 42 | Would rather be alone | 56E | Rashes, skin problems |
| 56D | Eye problems | 45 | Nervous, tense | 56F | Stomach aches |
| 75 | Shy, timid | 52 | Feels too guilty | 65 | Refuses to talk |
| 102 | Lacks energy | 54 | Overtired | 69 | Secretive |
| 103 | Unhappy, sad, depressed | 56B | Headaches | 71 | Self-conscious |
| 111 | Withdrawn | 56G | Vomiting | 91 | Thinks of suicide |
| 112 | Worries |  |  |  |  |
| Externalizing problems | | | | | |
| *Parcel 1* | | *Parcel 2* | | *Parcel 3* | |
| 3 | Argues | 16 | Mean | 28 | Breaks rules |
| 19 | Demands attention | 37 | Fights | 87 | Sudden mood changes |
| 20 | Destroys own things | 43 | Lies | 94 | Teases |
| 26 | Lacks guilt | 63 | Prefers older kids | 95 | Temper tantrums |
| 39 | Bad companions | 68 | Screams | 97 | Threatens |
| 57 | Attacks people | 81 | Steals at home^1^ | 104 | Loud |
| 21 | Destroys other's things | 82 | Steals outside home^1^ | 105 | Uses drugs |
| 101 | Truancy | 86 | Stubborn |  |  |
| Attention problems | | | | | |
| *Parcel 1* | | *Parcel 2* | | *Parcel 3* | |
| 4 | Fails to finish | 10 | Can't sit still | 17 | Daydreams |
| 8 | Can't concentrate | 13 | Confused | 41 | Impulsive |
|  |  | 61 | Poor school/ work functioning |  |  |

*Note.* *Item numbering as in the Youth Self-Report (YSR; Achenbach & Rescorla, 2001); YSR items 5, 28, 81/82, 105 and 4 correspond with Adult Self-Report (ASR; Rescorla & Achenbach, 2004) items 60, 23, 82, 6 and 59 respectively. ^1^Items combined such that the highest score was selected to match the ASR item 82 “I steal”.

**Table S2.** Longitudinal measurement invariance for self-reported internalizing, externalizing, and attention problems T1 - T6

| Model | | χ^2^ | *df* | RMSEA | CFI | TLI | SRMR | Model comparison | ΔRMSEA | ΔCFI | ΔSRMR | Decision |
| --- | --- | --- | --- | --- | --- | --- | --- | --- | --- | --- | --- | --- |
| 1 | Configural | 1732.500 | 1089 | .016 | .988 | .984 | .025 |  |  |  |  |  |
| 2 | Metric | 1881.1387 | 1119 | .017 | .985 | .981 | .027 | 2 vs 1 | .001 | -.003 | .002 | Accept |
| 3 | Scalar | 2843.790 | 1149 | .026 | .967 | .959 | .032 | 3 vs 2 | .009 | -.018 | .005 | Reject |
| 4a | Partial Scalar:  *Intercept EXT parcel 1 T1 freed* | 2652.430 | 1148 | .024 | .971 | .964 | .030 | 4a vs 2 | .007 | -.014 | .003 | Reject |
| 4b | Partial scalar:  *Intercepts EXT parcel 1 T1 and T6 freed* | 2572.449 | 1147 | .024 | .972 | .966 | .030 | 4b vs 2 | .007 | -.013 | .003 | Reject |
| 4c | Partial scalar:  *Intercepts EXT parcel 1 T1 and T6, ATT parcel 1 T3 freed* | 2503.324 | 1146 | .023 | .974 | .967 | .029 | 4c vs 2 | .006 | -.011 | .002 | Reject |
| 4d | Partial scalar:  *Intercepts EXT parcel 1 T1 and T6, ATT parcel 1 T3, INT parcel 2 T6 freed* | 2441.381 | 1145 | .023 | .975 | .969 | .029 | 4d vs 2 | .006 | -.010 | .002 | Reject |
| 4e | Partial scalar:  *Intercepts EXT parcel 1 T1 and T6, ATT parcel 1 T3, INT parcel 2 T5 and T6 freed* | 2404.699 | 1144 | .022 | .976 | .970 | .029 | 4e vs 2 | .005 | -.009 | .002 | Accept |

*Note.* INT = internalizing problems, EXT = externalizing problems, ATT= attention problems.

1. **Overview attempted multiple indicator and factor score RI-CLPM models**

**Table S3.** Overview specified multiple indicator and factor score RI-CLPM models with resulting errors

| Model | | Model details | Issues |
| --- | --- | --- | --- |
| **Multiple indicator RI-CLPMs** | |  |  |
| 1a | Partial scalar measurement model | As in Hamaker (2018), but with estimating residual covariances for the same items over time. | Standardized estimates larger than 1. |
| 1b | Partial scalar measurement model | As model 1a, but without estimating residual covariances for the same items over time. | Error: latent variable covariance matrix not positive definite, problem involving latent between factor of attention problems. |
| 1c | Partial scalar measurement model | As model 1a, but with specifying measurement factor variances to 1 and means to 0 at T1. | Error: no convergence, number of iterations exceeded.* |
| 1d | Partial scalar measurement model | As model 1c, but without freely estimating measurement factor covariances. | Error: standard errors could not be computed. |
| 1e | Partial scalar measurement model | As model 1a, but with default estimator instead of MLR. | Error: no convergence, number of iterations exceeded.* |
| 1f | Partial scalar measurement model | As model 1a, but with T1-T3 only. | Error: latent variable covariance matrix not positive definite, problem involving latent within internalizing problems T1. |
| 2a | Metric measurement model | As model 1a. | Standardized estimates larger than 1. |
| 2b | Metric measurement model | As model 1b. | Error: latent variable covariance matrix not positive definite, problem involving latent between factor of attention problems. |
| 2c | Metric measurement model | As model 1c. | Error: no convergence, number of iterations exceeded.* |
| 2d | Metric measurement model | As model 1c, but without freely estimating measurement factor covariances. | Error: standard errors could not be computed. |
| 3a | Configural measurement model | As model 1a. | Standardized estimates larger than 1. |
| 3b | Configural measurement model | As model 1b. | Standardized estimates larger than 1. |

**Table S3 continued.**

| **RI-CLPM with factor scores** | |  |  |
| --- | --- | --- | --- |
| 4a | Factor scores from partial scalar measurement model | Factor scores first saved from measurement invariance model, and then used in RI-CLPM. | Error: standard errors could not be computed. |
| 4b | Factor scores from partial scalar measurement model | As model 4a, but with default estimator instead of MLR. | Error: no convergence, number of iterations exceeded.* |
| 5a | Factor scores from metric measurement models for each variable separately | As 4a. | Error: latent variable covariance matrix not positive definite, problem involving latent within externalizing problems T6. |
| 5b | Factor scores from metric measurement models for each variable separately | As 4a, but factor means and variances freely estimated. | Error: standard errors not trustworthy; latent variable covariance matrix not positive definite, problem involving latent within externalizing T6. |
| 6 | Factor scores from configural measurement models for each variable separately | As 4a. | No errors, but bad model fit. |
| 7 | Factor scores with internalizing and externalizing problems only | As 4a. | Error: no convergence, number of iterations exceeded.* |

*Note.* *Increasing the number of iterations made no difference.

1. **Self-reported problems**

| Model | | χ^2^ | *df* | RMSEA | CFI | TLI | SRMR | Model comparison | ΔSBχ^2^,  *p*-value | ΔRMSEA | ΔCFI | ΔSRMR | Decision |
| --- | --- | --- | --- | --- | --- | --- | --- | --- | --- | --- | --- | --- | --- |
| 1 | Unconstrained model with fixed RI factor loadings | 351.139 | 84 | .038 | .981 | .965 | .045 |  |  |  |  |  |  |
| 2 | Grand means INT constrained | 615.715 | 89 | .052 | .962 | .934 | .056 | 2 vs 1 | 251.79, *p*<.001 | .052 | .962 | .056 | Reject |
| 3 | Grand means EXT constrained | 1302.706 | 89 | .078 | .912 | .849 | .103 | 3 vs 1 | 766.79, *p*<.001 | .078 | .912 | .103 | Reject |
| 4 | Grand means ATT constrained | 618.803 | 89 | .052 | .962 | .934 | .055 | 4 vs 1 | 279.01, *p*<.001 | .052 | .962 | .055 | Reject |
| 5 | Factor loadings bINT freed | 318.978 | 79 | .037 | .983 | .966 | .044 | 5 vs 1 | 33.35, *p*<.001 | -.037 | -.983 | -.044 | Accept |
| 6 | Factor loadings bINT + bEXT freed | 253.662 | 73 | .033 | .987 | .973 | .041 | 6 vs 5 | 61.13, *p*<.001 | .004 | -.004 | .003 | Accept |
| 7 | Factor loadings bINT + bEXT + bATT freed | 117.778 | 69 | .018 | .996 | .992 | .019 | 7 vs 6 | 158.95, *p*<.001 | .015 | -.009 | .022 | Accept |
| 8 | Model 7 + stability INT constrained | 138.416 | 73 | .020 | .995 | .990 | .022 | 8 vs 7 | 17.76, *p*=.001 | .002 | -.001 | .003 | Reject |
| 9 | Model 7 + stability EXT constrained | 145.74 | 73 | .021 | .995 | .989 | .022 | 9 vs 7 | 82.46, *p*<.001 | .003 | -.001 | .003 | Reject |
| 10 | Model 7 + stability ATT constrained | 141.831 | 73 | .021 | .995 | .990 | .021 | 10 vs 7 | 26.34, *p*<.001 | .003 | -.001 | .002 | Reject |
| 11 | Model 7 + within-wave INT - EXT constrained | 439.818 | 81 | .045 | .974 | .951 | .049 | 11 vs 7 | 310.27, *p*<.001 | .027 | -.022 | .030 | Reject |
| 12 | Model 7 + within-wave INT - ATT constrained | 193.254 | 81 | .025 | .992 | .985 | .029 | 12 vs 7 | 71.372, *p*<.001 | .007 | -.004 | .010 | Reject |
| 13 | Model 7 + within-wave EXT - ATT constrained | 326.518 | 81 | .037 | .982 | .966 | .043 | 13 vs 7 | 181.63, *p*<.001 | .019 | -.014 | .024 | Reject |
| 14 | Model 7 + cross-lagged INTt - EXTt+1 constrained | 130.716 | 73 | .019 | .996 | .991 | .021 | 14 vs 7 | 12.77, *p*=.012 | .001 | .000 | .002 | Reject |
| 15 | Model 7 + cross-lagged INTt - ATTt+1 constrained | 123.838 | 73 | .018 | .996 | .992 | .020 | 15 vs 7 | 6.07, *p*=.194 | .000 | .000 | .001 | Accept |
| 16 | Model 15 + cross-lagged EXTt - INTt+1 constrained | 128.422 | 77 | .017 | .996 | .993 | .020 | 16 vs 15 | 5.13, *p*=.275 | -.001 | .000 | .000 | Accept |
| 17 | Model 16 + cross-lagged EXTt - ATTt+1 constrained | 139.309 | 81 | .018 | .996 | .992 | .020 | 17 vs 16 | 10.53, *p*=.032 | .001 | .000 | .000 | Reject |
| 18 | Model 16 + cross-lagged ATTt - INTt+1 constrained | 140.724 | 81 | .018 | .996 | .992 | .021 | 18 vs 16 | 12.44, *p*=.014 | .001 | .000 | .001 | Reject |
| 19 | Model 16 + cross-lagged ATTt - EXTt+1 constrained | 140.977 | 81 | .018 | .996 | .992 | .021 | 19 vs 16 | 12.71, *p*=.013 | .001 | .000 | .001 | Reject |

**Table S4.** Model fit comparisons for RI-CLPMs with self-reported problems

*Note.* INT = internalizing problems, EXT = externalizing problems, ATT = attention problems, b = between-person factor

**Table S5.** Factor loadings random between-person factors self-reported problems

| Random factor | *B* | *β* | *SE* | *p* |
| --- | --- | --- | --- | --- |
| INT T1 | 1.00 | .42 | .02 | <.001 |
| INT T2 | 1.39 | .57 | .03 | <.001 |
| INT T3 | 1.93 | .77 | .03 | <.001 |
| INT T4 | 1.96 | .79 | .03 | <.001 |
| INT T5 | 1.82 | .70 | .03 | <.001 |
| INT T6 | 1.85 | .65 | .03 | <.001 |
|  |  |  |  |  |
| EXT T1 | 1.00 | .35 | .02 | <.001 |
| EXT T2 | 1.46 | .52 | .03 | <.001 |
| EXT T3 | 2.08 | .69 | .04 | <.001 |
| EXT T4 | 2.03 | .80 | .03 | <.001 |
| EXT T5 | 1.52 | .71 | .04 | <.001 |
| EXT T6 | 1.49 | .71 | .03 | <.001 |
|  |  |  |  |  |
| ATT T1 | 1.00 | .38 | .02 | <.001 |
| ATT T2 | 1.55 | .53 | .03 | <.001 |
| ATT T3 | 2.27 | .74 | .04 | <.001 |
| ATT T4 | 2.45 | .80 | .03 | <.001 |
| ATT T5 | 2.04 | .67 | .04 | <.001 |
| ATT T6 | 1.83 | .60 | .04 | <.001 |

*Note.* INT = internalizing problems, EXT = externalizing problems, ATT = attention problems.

**Table S6.** Results from the final constrained RI-CLPM with self-reported problems (model 16)

|  | INT | | | | EXT | | | | ATT | | | |  |
| --- | --- | --- | --- | --- | --- | --- | --- | --- | --- | --- | --- | --- | --- |
|  | *β* | *SE* | *p* | [95% CI] | *β* | *SE* | *p* | [95% CI] | *β* | *SE* | *p* | [95% CI] | |
| *Between-person correlations* |  |  |  |  |  |  |  |  |  |  |  |  | |
| INT - EXT/ ATT |  |  |  |  | .57 | .03 | <.001 | [.52, .61] | .66 | .02 | <.001 | [.63, .70] | |
| EXT - ATT |  |  |  |  |  |  |  |  | .73 | .02 | <.001 | [.70, .76] | |
| *Within-wave (residual) correlations* |  |  |  |  |  |  |  |  |  |  |  |  | |
| INT - EXT / ATT T1 |  |  |  |  | .53 | .02 | <.001 | [.50, .56] | .56 | .02 | <.001 | [.53, .59] | |
| EXT - ATT T1 |  |  |  |  |  |  |  |  | .54 | .02 | <.001 | [.51, .57] | |
| INT - EXT / ATT T2 |  |  |  |  | .40 | .02 | <.001 | [.36, .43] | .46 | .02 | <.001 | [.42, .49] | |
| EXT - ATT T2 |  |  |  |  |  |  |  |  | .50 | .02 | <.001 | [.46, .53] | |
| INT - EXT / ATT T3 |  |  |  |  | .29 | .04 | <.001 | [.24, .35] | .31 | .04 | <.001 | [.24, .38] | |
| EXT - ATT T3 |  |  |  |  |  |  |  |  | .44 | .03 | <.001 | [38, .49] | |
| INT - EXT / ATT T4 |  |  |  |  | .39 | .05 | <.001 | [.31, .47] | .39 | .05 | <.001 | [.31, .48] | |
| EXT - ATT T4 |  |  |  |  |  |  |  |  | .38 | .06 | <.001 | [.27, .48] | |
| INT - EXT / ATT T5 |  |  |  |  | .54 | .03 | <.001 | [.49, .59] | .49 | .03 | <.001 | [.43, .54] | |
| EXT - ATT T5 |  |  |  |  |  |  |  |  | .45 | .04 | <.001 | [.39, .51] | |
| INT - EXT / ATT T6 |  |  |  |  | .55 | .03 | <.001 | [.50, .59] | .50 | .03 | <.001 | [.45, .54] | |
| EXT - ATT T6 |  |  |  |  |  |  |  |  | .48 | .03 | <.001 | [.43, .52] | |
| *Lagged paths* |  |  |  |  |  |  |  |  |  |  |  |  | |
| INT T1 - INT/ EXT/ ATT^a^ T2 | .33 | .03 | <.001 | [.28, .39] | .03 | .03 | .289 | [-.01, .09] | .00 | .02 | .876 | [-.04, .03] | |
| INT T2 - INT/ EXT/ ATT^a^ T3 | .29 | .05 | <.001 | [.21, .37] | -.06 | .03 | .076 | [-.12, -.00] | .00 | .02 | .875 | [-.04, .03] | |
| INT T3 - INT/ EXT/ ATT^a^ T4 | .04 | .10 | .671 | [-.12, .20] | -.02 | .05 | .754 | [-.10, .07] | .00 | .02 | .876 | [-.04, .03] | |
| INT T4 - INT/ EXT/ ATT^a^ T5 | .13 | .07 | .058 | [.02, .25] | .04 | .05 | .407 | [-.04, .12] | .00 | .02 | .876 | [-.03, .02] | |
| INT T5 - INT/ EXT/ ATT^a^ T6 | .34 | .05 | <.001 | [.25, .42] | .10 | .05 | .037 | [.02, .19] | .00 | .02 | .876 | [-.03, .03] | |
|  |  |  |  |  |  |  |  |  |  |  |  |  | |
| EXT T1 - INT^a^/ EXT/ ATT T2 | -.06 | .02 | .011 | [-.09, -.02] | .29 | .03 | <.001 | [.24, .34] | .12 | .03 | <.001 | [.08, .17] | |
| EXT T2 - INT^a^/ EXT/ ATT T3 | -.06 | .02 | .009 | [-.10, -.02] | .25 | .05 | <.001 | [.17, .33] | .02 | .04 | .710 | [-.05, .08] | |
| EXT T3 - INT^a^/ EXT/ ATT T4 | -.06 | .03 | .013 | [-.10, -.02] | .05 | .09 | .563 | [-.09, .19] | .01 | .06 | .882 | [-.09, .11] | |
| EXT T4 - INT^a^/ EXT/ ATT T5 | -.04 | .01 | .012 | [-.06, -.01] | .04 | .08 | .632 | [-.09, .17] | -.04 | .05 | .396 | [-.13, .04] | |
| EXT T5 - INT^a^/ EXT/ ATT T6 | -.03 | .01 | .012 | [-.05, -.01] | .10 | .08 | .211 | [-.03, .24] | .05 | .04 | .220 | [-.02, .11] | |
|  |  |  |  |  |  |  |  |  |  |  |  |  | |
| ATT T1 - INT/ EXT/ ATT T2 | .07 | .03 | .015 | [.02, .12] | .04 | .03 | .225 | [-.02, .08] | .22 | .03 | <.001 | [.17, .27] | |
| ATT T2 - INT/ EXT/ ATT T3 | .01 | .04 | .840 | [-.05, .01] | .11 | .04 | .004 | [.05, .17] | .21 | .05 | <.001 | [.12, .30] | |
| ATT T3 - INT/ EXT/ ATT T4 | -.12 | .06 | .054 | [-.22, -.02] | -.08 | .07 | .268 | [-.19, .04] | -.08 | .10 | .403 | [-.25, .08] | |
| ATT T4 - INT/ EXT/ ATT T5 | -.07 | .07 | .316 | [-.17, .04] | -.07 | .07 | .305 | [-.17, .04] | .12 | .08 | .151 | [-.02, .25] | |
| ATT T5 - INT/ EXT/ ATT T6 | .05 | .05 | .247 | [-.02, .13] | .05 | .05 | .359 | [-.04, .13] | .34 | .05 | <.001 | [.25, .42] | |

*Note.* INT = internalizing problems, EXT = externalizing problems, ATT = attention problems. ^a^Paths constrained to be equal over time.

1. **Sex differences**

**Table S7.** Model fit comparisons for RI-CLPMs with self-reported problems of males versus females

| Model | | χ^2^ | *df* | RMSEA | CFI | TLI | SRMR | Model comparison | ΔSBχ^2^, *p*-value | ΔRMSEA | ΔCFI | ΔSRMR | Decision |
| --- | --- | --- | --- | --- | --- | --- | --- | --- | --- | --- | --- | --- | --- |
| 1 | Unconstrained model* | 228.505 | 169 | .018 | .996 | .992 | .029 |  |  |  |  |  |  |
| 2 | Constrained model | 380.115 | 227 | .025 | .989 | .985 | .045 | 2 vs 1 | 145.35, *p*<.001 | -.007 | -.007 | .016 | Reject |
| 3 | Between-person EXT-ATT freed | 360.154 | 226 | .023 | .990 | .987 | .047 | 3 vs 1 | 126.87, *p*<.001 | -.006 | -.005 | .018 | Reject |
| 4 | Model 3 + LINT_T2_ - LATT_T2_ freed | 345.809 | 225 | .022 | .991 | .988 | .047 | 4 vs 1 | 113.62, *p*<.001 | -.005 | -.004 | .018 | Reject |
| 5 | Model 4 + LEXT_T6_ - LATT_T6_ freed | 334.605 | 224 | .021 | .992 | .989 | .047 | 5 vs 1 | 103.24, *p*<.001 | -.004 | -.003 | .018 | Reject |
| 6 | Model 5 + all within-wave INT - ATT freed | 315.075 | 219 | .020 | .993 | .990 | .046 | 6 vs 1 | 84.80, *p*=.002 | -.003 | -.002 | .017 | Reject |
| 7 | Model 6 + all within-wave EXT - ATT freed | 298.949 | 214 | .019 | .994 | .991 | .044 | 7 vs 1 | 69.51, *p*=.011 | -.002 | -.001 | .015 | Reject |
| 8 | Model 7 + all within-wave INT - EXT freed | 280.595 | 208 | .018 | .995 | .992 | .042 | 8 vs 1 | 52.13, *p*=.078 | -.001 | .000 | .013 | Accept |

*Note. **With time constraints separate for males and females as in RI-CLPM 16 from the main analyses; INT = internalizing problems, EXT = externalizing problems, ATT = attention problems

**Table S8A.** Results from final constrained RI-CLPM with self-reported problems for males (model 8)

|  | INT | | | | EXT | | | | ATT | | | | |
| --- | --- | --- | --- | --- | --- | --- | --- | --- | --- | --- | --- | --- | --- |
|  | *β* | *SE* | *p* | [95% CI] | *β* | *SE* | *p* | [95% CI] | *β* | *SE* | *p* | [95% CI] |  |
| *Between-person correlations* |  |  |  |  |  |  |  |  |  |  |  |  |  |
| INT - EXT^a^/ ATT^a^ |  |  |  |  | .65 | .03 | <.001 | [.60, .70] | .73 | .03 | <.001 | [.68, .80] |  |
| EXT - ATT |  |  |  |  |  |  |  |  | .73 | .02 | <.001 | [.70, .77] |  |
| *Within-wave correlations* |  |  |  |  |  |  |  |  |  |  |  |  |  |
| INT - EXT / ATT T1 |  |  |  |  | .57 | .03 | <.001 | [.53, .61] | .59 | .02 | <.001 | [.55, .63] |  |
| EXT - ATT T1 |  |  |  |  |  |  |  |  | .57 | .03 | <.001 | [.53, .61] |  |
| INT - EXT / ATT T2 |  |  |  |  | .42 | .03 | <.001 | [.37, .47] | .43 | .03 | <.001 | [.38, .48] |  |
| EXT - ATT T2 |  |  |  |  |  |  |  |  | .51 | .03 | <.001 | [.46, .56] |  |
| INT - EXT / ATT T3 |  |  |  |  | .28 | .04 | <.001 | [.20, .35] | .25 | .05 | <.001 | [.17, .43] |  |
| EXT - ATT T3 |  |  |  |  |  |  |  |  | .42 | .04 | <.001 | [.35, .49] |  |
| INT - EXT / ATT T4 |  |  |  |  | .35 | .07 | <.001 | [.24, .46] | .33 | .08 | <.001 | [.21, .46] |  |
| EXT - ATT T4 |  |  |  |  |  |  |  |  | .42 | .06 | <.001 | [.31, .52] |  |
| INT - EXT / ATT T5 |  |  |  |  | .53 | .05 | <.001 | [.45, .61] | .51 | .05 | <.001 | [.42, .59] |  |
| EXT - ATT T5 |  |  |  |  |  |  |  |  | .45 | .05 | <.001 | [.37, .54] |  |
| INT - EXT / ATT T6 |  |  |  |  | .49 | .05 | <.001 | [.42, .57] | .51 | .04 | <.001 | [.44, .57] |  |
| EXT - ATT T6 |  |  |  |  |  |  |  |  | .48 | .05 | <.001 | [.41, .56] |  |
| *Lagged paths* |  |  |  |  |  |  |  |  |  |  |  |  |  |
| INT T1 - INT^a^/ EXT^a^/ ATT^ab^ T2 | .37 | .03 | <.001 | [.31, .42] | .03 | .03 | .283 | [.00, .04] | -.01 | .02 | .737 | [-.04, .03] |  |
| INT T2 - INT^a^/ EXT^a^/ ATT^ab^ T3 | .31 | .05 | <.001 | [.23, .38] | -.05 | .03 | .093 | [.00, .04] | -.01 | .02 | .736 | [-.04, .02] |  |
| INT T3 - INT^a^/ EXT^a^/ ATT^ab^ T4 | .09 | .10 | .342 | [-.07, .25] | -.01 | .04 | .783 | [.00, .05] | -.01 | .02 | .737 | [-.03, .02] |  |
| INT T4 - INT^a^/ EXT^a^/ ATT^ab^ T5 | .11 | .06 | .048 | [.02, .21] | .01 | .04 | .820 | [.00, .06] | .00 | .01 | .737 | [-.03, .02] |  |
| INT T5 - INT^a^/ EXT^a^/ ATT^ab^ T6 | .34 | .06 | <.001 | [.24, .45] | .10 | .05 | .025 | [.00, .08] | -.01 | .02 | .737 | [-.03, .02] |  |
|  |  |  |  |  |  |  |  |  |  |  |  |  |  |
| EXT T1 - INT^ab^/ EXT^a^/ ATT^a^ T2 | -.04 | .03 | .136 | [-.09, .00] | .30 | .03 | <.001 | [.25, .35] | .14 | .03 | <.001 | [.09, .19] |  |
| EXT T2 - INT^ab^/ EXT^a^/ ATT^a^ T3 | -.04 | .03 | .135 | [-.09, .00] | .27 | .05 | <.001 | [.20, .35] | .03 | .04 | .422 | [-.03, .10] |  |
| EXT T3 - INT^ab^/ EXT^a^/ ATT^a^ T4 | -.05 | .03 | .139 | [-.10, .01] | .13 | .07 | .069 | [.01, .25] | .05 | .06 | .401 | [-.05, .14] |  |
| EXT T4 - INT^ab^/ EXT^a^/ ATT^a^ T5 | -.02 | .02 | .134 | [-.05, .00] | .07 | .08 | .351 | [-.05, .20] | -.04 | .05 | .414 | [-.13, .04] |  |
| EXT T5 - INT^ab^/ EXT^a^/ ATT^a^ T6 | -.02 | .01 | .137 | [-.04, .00] | .08 | .08 | .359 | [-.06, .21] | .05 | .04 | .238 | [-.02, .11] |  |
|  |  |  |  |  |  |  |  |  |  |  |  |  |  |
| ATT T1 - INT^a^/ EXT^a^/ ATT^a^ T2 | .07 | .03 | .024 | [.02, .13] | .04 | .03 | .183 | [-.01, .09] | .23 | .03 | .008 | [.18, .28] |  |
| ATT T2 - INT^a^/ EXT^a^/ ATT^a^ T3 | .00 | .04 | .971 | [-.06, .07] | .11 | .04 | .002 | [.05, .17] | .24 | .05 | <.001 | [.15, .32] |  |
| ATT T3 - INT^a^/ EXT^a^/ ATT^a^ T4 | -.16 | .07 | .036 | [-.28, -.03] | -.05 | .06 | .343 | [-.15, .04] | -.04 | .10 | .705 | [-.20, .12] |  |
| ATT T4 - INT^a^/ EXT^a^/ ATT^a^ T5 | -.09 | .08 | .254 | [-.23, .04] | -.08 | .06 | .194 | [-.19, .02] | .08 | .09 | .416 | [-.08, .23] |  |
| ATT T5 - INT^a^/ EXT^a^/ ATT^a^ T6 | .04 | .06 | .466 | [-.05, .13] | .01 | .06 | .934 | [.-.09, .10] | .29 | .06 | <.001 | [.19, .40] |  |

*Note.* INT = internalizing problems, EXT = externalizing problems, ATT = attention problems. ^a^Paths constrained to be equal for males and females; ^b^Paths constrained to be equal over time.

**Table S8B.** Results from final constrained RI-CLPM with self-reported problems for females (model 8)

|  | INT | | | | EXT | | | | ATT | | | | |
| --- | --- | --- | --- | --- | --- | --- | --- | --- | --- | --- | --- | --- | --- |
|  | *β* | *SE* | *p* | [95% CI] | *β* | *SE* | *p* | [95% CI] | *β* | *SE* | *p* | [95% CI] |  |
| *Between-person correlations* |  |  |  |  |  |  |  |  |  |  |  |  |  |
| INT - EXT^a^/ ATT^a^ |  |  |  |  | .66 | .03 | <.001 | [.62, .71] | .69 | .03 | <.001 | [.65, .73] |  |
| EXT - ATT |  |  |  |  |  |  |  |  | .74 | .03 | <.001 | [.70, .78] |  |
| *Within-wave correlations* |  |  |  |  |  |  |  |  |  |  |  |  |  |
| INT - EXT / ATT T1 |  |  |  |  | .52 | .03 | <.001 | [.47, .57] | .53 | .03 | <.001 | [.49, .57] |  |
| EXT - ATT T1 |  |  |  |  |  |  |  |  | .51 | .02 | <.001 | [.47, .55] |  |
| INT - EXT / ATT T2 |  |  |  |  | .38 | .03 | <.001 | [.33, .44] | .47 | .03 | <.001 | [.42, .52] |  |
| EXT - ATT T2 |  |  |  |  |  |  |  |  | .49 | .03 | <.001 | [.44, .53] |  |
| INT - EXT / ATT T3 |  |  |  |  | .33 | .04 | <.001 | [.26, .40] | .37 | .05 | <.001 | [.29, .44] |  |
| EXT - ATT T3 |  |  |  |  |  |  |  |  | .48 | .03 | <.001 | [.43, .54] |  |
| INT - EXT / ATT T4 |  |  |  |  | .42 | .06 | <.001 | [.33, .51] | .43 | .05 | <.001 | [.34, .51] |  |
| EXT - ATT T4 |  |  |  |  |  |  |  |  | .38 | .07 | <.001 | [.27, .49] |  |
| INT - EXT / ATT T5 |  |  |  |  | .54 | .04 | <.001 | [.47, .61] | .47 | .05 | <.001 | [.39, .54] |  |
| EXT - ATT T5 |  |  |  |  |  |  |  |  | .40 | .06 | <.001 | [.30, .50] |  |
| INT - EXT / ATT T6 |  |  |  |  | .60 | .03 | <.001 | [.54, .65] | .50 | .03 | <.001 | [.46, .57] |  |
| EXT - ATT T6 |  |  |  |  |  |  |  |  | .46 | .03 | <.001 | [.40, .51] |  |
| *Lagged paths* |  |  |  |  |  |  |  |  |  |  |  |  |  |
| INT T1 - INT^a^/ EXT^a^/ ATT^ab^ T2 | .31 | .03 | <.001 | [.26, .36] | .03 | .03 | .283 | [-.02 .09] | -.01 | .02 | .737 | [-.04, .03] |  |
| INT T2 - INT^a^/ EXT^a^/ ATT^ab^ T3 | .29 | .04 | <.001 | [.22, .36] | -.06 | .04 | .087 | [-.12, .00] | -.01 | .02 | .736 | [-.04, .03] |  |
| INT T3 - INT^a^/ EXT^a^/ ATT^ab^ T4 | .08 | .09 | .355 | [-.07, .23] | -.01 | .05 | .784 | [-.09, .07] | -.01 | .02 | .737 | [-.04, .03] |  |
| INT T4 - INT^a^/ EXT^a^/ ATT^ab^ T5 | .14 | .07 | .044 | [.03, .25] | .01 | .06 | .820 | [-.08, .11] | -.01 | .02 | .737 | [-.04, .02] |  |
| INT T5 - INT^a^/ EXT^a^/ ATT^ab^ T6 | .33 | .06 | <.001 | [.24, .42] | .13 | .06 | .019 | [.04, .22] | -.01 | .02 | .737 | [-.04, .02] |  |
|  |  |  |  |  |  |  |  |  |  |  |  |  |  |
| EXT T1 - INT^ab^/ EXT^a^/ ATT^a^ T2 | -.03 | .02 | .136 | [-.06, .00] | .26 | .03 | <.001 | [.22, .31] | .11 | .03 | <.001 | [.0, .15] |  |
| EXT T2 - INT^ab^/ EXT^a^/ ATT^a^ T3 | -.03 | .02 | .136 | [-.06, .00] | .26 | .05 | <.001 | [.19, .34] | .03 | .04 | .424 | [-.03, .09] |  |
| EXT T3 - INT^ab^/ EXT^a^/ ATT^a^ T4 | -.03 | .02 | .136 | [-.07, .00] | .13 | .07 | .073 | [.01, .25] | .04 | .05 | .407 | [-.04, .13] |  |
| EXT T4 - INT^ab^/ EXT^a^/ ATT^a^ T5 | -.03 | .02 | .136 | [-.04, .00] | .07 | .07 | .339 | [-.05, .19] | -.04 | .05 | .416 | [-.12, .04] |  |
| EXT T5 - INT^ab^/ EXT^a^/ ATT^a^ T6 | -.03 | .02 | .136 | [-.03, .00] | .08 | .08 | .363 | [-.06, .21] | .04 | .04 | .248 | [-.02, .11] |  |
|  |  |  |  |  |  |  |  |  |  |  |  |  |  |
| ATT T1 - INT^a^/ EXT^a^/ ATT^a^ T2 | .06 | .03 | .024 | [.02, .10] | .04 | .03 | .181 | [-.01, .09] | .22 | .03 | .008 | [.17, .27] |  |
| ATT T2 - INT^a^/ EXT^a^/ ATT^a^ T3 | .00 | .03 | .971 | [-.05, .05] | .12 | .04 | .002 | [.06 .18] | .23 | .05 | <.001 | [.15, .31] |  |
| ATT T3 - INT^a^/ EXT^a^/ ATT^a^ T4 | -.12 | .05 | .033 | [-.20, -.03] | -.06 | .06 | .351 | [-.17, .05] | -.04 | .10 | .706 | [-.20, .12] |  |
| ATT T4 - INT^a^/ EXT^a^/ ATT^a^ T5 | -.08 | .07 | .259 | [-.20, .04] | -.09 | .07 | .209 | [-.21, .03] | .08 | .09 | .412 | [-.08, .23] |  |
| ATT T5 - INT^a^/ EXT^a^/ ATT^a^ T6 | .03 | .05 | .470 | [-.04, .11] | .01 | .06 | .934 | [-.10, .11] | .30 | .07 | <.001 | [.20, .41] |  |

*Note.* INT = internalizing problems, EXT = externalizing problems, ATT= attention problems. ^a^Paths constrained to be equal for males and females; ^b^Paths constrained to be equal over time.

1. **Sensitivity analyses**

**Table S9.** Model fit comparisons for RI-CLPMs with self-reported problems controlling for SES, mental healthcare, and medication use

| Model | | χ^2^ | *df* | RMSEA | CFI | TLI | SRMR | Model comparison | ΔSBχ^2^, | ΔRMSEA | ΔCFI | ΔSRMR | Decision |
| --- | --- | --- | --- | --- | --- | --- | --- | --- | --- | --- | --- | --- | --- |
|  |  |  |  |  |  |  |  |  | *p*-value |  |  |  |  |
| 1 | Unconstrained model^1^ | 318.694 | 84 | .035 | .984 | .961 | .032 |  |  |  |  |  |  |
| 2 | Grand means constrained INT | 464.782 | 89 | .044 | .974 | .940 | .045 | 2 vs 1 | 197.30, *p*<.001 | .009 | -.010 | -.021 | Reject |
| 3 | Grand means constrained EXT | 679.025 | 89 | .055 | .960 | .906 | .062 | 3 vs 1 | 420.88, *p*<.001 | .020 | -.024 | -.055 | Reject |
| 4 | Grand means constrained ATT | 409.867 | 89 | .040 | .978 | .949 | .039 | 4 vs 1 | 111.20, *p*<.001 | .005 | -.006 | -.012 | Reject |
| 5 | Factor loadings bINT freed | 285.486 | 79 | .034 | .986 | .963 | .030 | 1 vs 5 | 33.60, *p*<.001 | -.001 | .002 | .002 | Accept |
| 6 | Factor loadings bINT + bEXT freed | 215.55 | 73 | .029 | .990 | .973 | .028 | 5 vs 6 | 66.83, *p*<.001 | -.005 | .004 | .010 | Accept |
| 7 | Factor loadings bINT + bEXT + bATT freed | 114.572 | 69 | .017 | .997 | .991 | .016 | 7 vs 6 | 131.24, *p*<.001 | -.012 | .007 | .018 | Accept |
| 8 | Model 7 + stability INT constrained | 131.521 | 73 | .019 | .996 | .989 | .018 | 8 vs 7 | 14.99, *p*<.001 | .002 | -.001 | -.002 | Reject |
| 9 | Model 7 + stability EXT constrained | 147.793 | 73 | .021 | .995 | .986 | .017 | 9 vs 7 | n.a.* | .004 | -.002 | -.005 | Reject |
| 10 | Model 7 + stability ATT constrained | 138.85 | 73 | .020 | .996 | .987 | .018 | 10 vs 7 | 28.83, *p*<.001 | .003 | -.001 | -.004 | Reject |
| 11 | Model 7 + within-wave INT - EXT constrained | 426.986 | 81 | .044 | .976 | .940 | .039 | 11 vs 7 | 370.33, *p*<.001 | .027 | -.021 | -.051 | Reject |
| 12 | Model 7 + within-wave INT - ATT constrained | 183.434 | 81 | .024 | .993 | .982 | .024 | 12 vs 7 | 67.09, *p*<.001 | .007 | -.004 | -.009 | Reject |
| 13 | Model 7 + within-wave EXT - ATT constrained | 319.657 | 81 | .036 | .984 | .958 | .035 | 13 vs 7 | 189.93, *p*<.001 | .019 | -.013 | -.033 | Reject |
| 14 | Model 7 + cross-lagged INTt - EXTt+1 constrained | 124.731 | 73 | .018 | .996 | .990 | .018 | 14 vs 7 | 10.07, *p*=.039 | .001 | -.001 | -.001 | Reject |
| 15 | Model 7 + cross-lagged INTt - ATTt+1 constrained | 117.937 | 73 | .017 | .997 | .991 | .017 | 15 vs 7 | 3.42, *p*=.489 | .000 | .000 | .000 | Accept |
| 16 | Model 15 + cross-lagged EXTt - INTt+1 constrained | 122.942 | 77 | .016 | .997 | .992 | .017 | 16 vs 15 | 5.31, *p*=.257 | -.001 | .000 | .001 | Accept |
| 17 | Model 16 + cross-lagged EXTt - ATTt+1 constrained | 131.488 | 81 | .017 | .997 | .991 | .017 | 17 vs 16 | 8.37, *p*=.079 | .001 | .000 | -.001 | Accept |
| 18 | Model 16 + cross-lagged ATTt - INTt+1 constrained | 145.594 | 85 | .018 | .996 | .990 | .018 | 18 vs 17 | 14.64, *p*=.006 | .001 | -.001 | -.001 | Reject |
| 19 | Model 16 + cross-lagged ATTt - EXTt+1 constrained | 142.092 | 85 | .017 | .996 | .991 | .018 | 19 vs 17 | 10.85, *p*=.028 | .000 | -.001 | .000 | Reject |

*Note.* ^1^with fixed factor loadings for the random intercepts; INT = internalizing problems, EXT = externalizing problems, ATT = attention problems, b = between-person factor. *not available; difference testing resulted in a negative difference due to a low MLR scaling factor in model 9, which is most likely due to the absence of within-person stability from T3 onwards for externalizing problems.

|  | INT | | | | EXT | | | | ATT | | | |
| --- | --- | --- | --- | --- | --- | --- | --- | --- | --- | --- | --- | --- |
|  | *β* | *SE* | *p* | [95% CI] | *β* | *SE* | *p* | [95% CI] | *β* | *SE* | *p* | [95% CI] |
| *Between-person correlations* |  |  |  |  |  |  |  |  |  |  |  |  |
| INT - EXT/ ATT |  |  |  |  | .46 | .03 | <.001 | [.40, .51] | .58 | .03 | <.001 | [.53, .62] |
| EXT - ATT |  |  |  |  |  |  |  |  | .64 | .02 | <.001 | [.60, .68] |
| *Within-wave (residual) correlations* |  |  |  |  |  |  |  |  |  |  |  |  |
| INT - EXT/ ATT T1 |  |  |  |  | .54 | .02 | <.001 | [.51, .57] | .56 | .02 | <.001 | [.53, .59] |
| EXT - ATT T1 |  |  |  |  |  |  |  |  | .53 | .02 | <.001 | [.50, .56] |
| INT - EXT/ ATT T2 |  |  |  |  | .40 | .02 | <.001 | [.37, .44] | .47 | .02 | <.001 | [.43, .50] |
| EXT - ATT T2 |  |  |  |  |  |  |  |  | .50 | .02 | <.001 | [.47, .53] |
| INT - EXT/ ATT T3 |  |  |  |  | .30 | .04 | <.001 | [.25, .36] | .33 | .04 | <.001 | [.26, .40] |
| EXT - ATT T3 |  |  |  |  |  |  |  |  | .46 | .03 | <.001 | [.41, .51] |
| INT - EXT/ ATT T4 |  |  |  |  | .42 | .04 | <.001 | [.35, .50] | .43 | .05 | <.001 | [.36, .51] |
| EXT - ATT T4 |  |  |  |  |  |  |  |  | .44 | .06 | <.001 | [.35, .53] |
| INT - EXT/ ATT T5 |  |  |  |  | .53 | .03 | <.001 | [.48, .58] | .47 | .03 | <.001 | [.42, .52] |
| EXT - ATT T5 |  |  |  |  |  |  |  |  | .46 | .03 | <.001 | [.41, .52] |
| INT - EXT/ ATT T6 |  |  |  |  | .54 | .03 | <.001 | [.49, .58] | .49 | .03 | <.001 | [.44, .53] |
| EXT - ATT T6 |  |  |  |  |  |  |  |  | .47 | .03 | <.001 | [.42, .51] |
| *Lagged paths* |  |  |  |  |  |  |  |  |  |  |  |  |
| INT T1 - INT/ EXT/ ATT^a^ T2 | .30 | .04 | <.001 | [.24, .36] | .03 | .03 | .275 | [-.02, .08] | .00 | .02 | .963 | [-.03, .03] |
| INT T2 - INT/ EXT/ ATT^a^ T3 | .25 | .06 | <.001 | [.15, .34] | -.06 | .04 | .109 | [-.11, .001] | .00 | .02 | .963 | [-.04, .04] |
| INT T3 - INT/ EXT/ ATT^a^ T4 | .06 | .09 | .506 | [-.09, .20] | .00 | .05 | .947 | [-.09, .08] | .00 | .02 | .963 | [-.03, .03] |
| INT T4 - INT/ EXT/ ATT^a^ T5 | .20 | .06 | .002 | [.09, .30] | .07 | .05 | .187 | [-.02, .15] | .00 | .02 | .963 | [-.03, .03] |
| INT T5 - INT/ EXT/ ATT^a^ T6 | .35 | .05 | <.001 | [.28, .43] | .09 | .05 | .057 | [.01, .17] | .00 | .02 | .963 | [-.03, .03] |
|  |  |  |  |  |  |  |  |  |  |  |  |  |
| EXT T1 - INT^a^/ EXT/ ATT^a^ T2 | -.05 | .02 | .053 | [-.09, -.01] | .27 | .03 | <.001 | [.22, .31] | .07 | .02 | .002 | [.03, .11] |
| EXT T2 - INT^a^/ EXT/ ATT^a^ T3 | -.05 | .03 | .048 | [-.10, -.01] | .26 | .05 | <.001 | [.18, .34] | .08 | .03 | .003 | [.03, .12] |
| EXT T3 - INT^a^/ EXT/ ATT^a^ T4 | -.05 | .03 | .060 | [-.09, -.01] | .04 | .09 | .640 | [-.11, .19] | .08 | .03 | .003 | [.03, .12] |
| EXT T4 - INT^a^/ EXT/ ATT^a^ T5 | -.03 | .02 | .055 | [-.05, .00] | .05 | .08 | .509 | [-.08, .19] | .04 | .02 | .005 | [.02, .07] |
| EXT T5 - INT^a^/ EXT/ ATT^a^ T6 | -.02 | .01 | .056 | [-.04, -.09] | .11 | .08 | .199 | [-.03, .24] | .04 | .01 | .002 | [.02, .06] |
|  |  |  |  |  |  |  |  |  |  |  |  |  |
| ATT T1 - INT/ EXT/ ATT T2 | .09 | .03 | .003 | [.04, .14] | .05 | .03 | .084 | [.00, .10] | .25 | .03 | <.001 | [.21, .30] |
| ATT T2 - INT/ EXT/ ATT T3 | .03 | .04 | .508 | [-.04, .09] | .10 | .04 | .006 | [.04, .16] | .17 | .06 | .002 | [.08, .26] |
| ATT T3 - INT/ EXT/ ATT T4 | -.09 | .06 | .132 | [-.19, .01] | -.04 | .07 | .586 | [-.15, .07] | -.18 | .12 | .134 | [-.39, .02] |
| ATT T4 - INT/ EXT/ ATT T5 | -.10 | .06 | .091 | [-.19, .00] | -.08 | .06 | .206 | [-.18, .02] | .08 | .09 | .363 | [-.06, .22] |
| ATT T5 - INT/ EXT/ ATT T6 | .02 | .04 | .558 | [-.04, .09] | .06 | .04 | .172 | [-.01, .13] | .34 | .05 | <.001 | [.26, .41] |

**Table S10.** Results from the final constrained RI-CLPM with self-reported problems controlling for SES, mental healthcare, and medication use (model 17)

*Note*. INT = internalizing problems, EXT = externalizing problems, ATT = attention problems. ^a^Paths constrained to be equal over time.

**Table S11A.** Results from the final constrained RI-CLPM with self-reported problems for males controlling for SES, mental healthcare, and medication use

|  | INT | | | | EXT | | | | ATT | | | |
| --- | --- | --- | --- | --- | --- | --- | --- | --- | --- | --- | --- | --- |
|  | *β* | *SE* | *p* | [95% CI] | *β* | *SE* | *p* | [95% CI] | *β* | *SE* | *p* | [95% CI] |
| *Between-person correlations* |  |  |  |  |  |  |  |  |  |  |  |  |
| INT - EXT/ ATT |  |  |  |  | .55 | .04 | <.001 | [.49, .61] | .65 | .03 | <.001 | [.59, .70] |
| EXT - ATT |  |  |  |  |  |  |  |  | .66 | .03 | <.001 | [.61, .71] |
| *Within-wave (residual) correlations* |  |  |  |  |  |  |  |  |  |  |  |  |
| INT - EXT/ ATT T1 |  |  |  |  | .57 | .03 | <.001 | [.53, .61] | .59 | .02 | <.001 | [.55, .63] |
| EXT - ATT T1 |  |  |  |  |  |  |  |  | .57 | .03 | <.001 | [.53, .61] |
| INT - EXT/ ATT T2 |  |  |  |  | .43 | .03 | <.001 | [.38, .48] | .43 | .03 | <.001 | [.39, .48] |
| EXT - ATT T2 |  |  |  |  |  |  |  |  | .51 | .03 | <.001 | [.46, .56] |
| INT - EXT/ ATT T3 |  |  |  |  | .27 | .04 | <.001 | [.20, .34] | .26 | .05 | <.001 | [.17, .34] |
| EXT - ATT T3 |  |  |  |  |  |  |  |  | .42 | .04 | <.001 | [.36, .49] |
| INT - EXT/ ATT T4 |  |  |  |  | .37 | .07 | <.001 | [.26, .48] | .36 | .08 | <.001 | [.23, .48] |
| EXT - ATT T4 |  |  |  |  |  |  |  |  | .44 | .07 | <.001 | [.33, .54] |
| INT - EXT/ ATT T5 |  |  |  |  | .54 | .05 | <.001 | [.46, .61] | .50 | .05 | <.001 | [.42, .58] |
| EXT - ATT T5 |  |  |  |  |  |  |  |  | .46 | .05 | <.001 | [.37, .54] |
| INT - EXT/ ATT T6 |  |  |  |  | .49 | .05 | <.001 | [.41, .57] | .49 | .04 | <.001 | [.42, .56] |
| EXT - ATT T6 |  |  |  |  |  |  |  |  | .48 | .05 | <.001 | [.40, .55] |
| *Lagged paths* |  |  |  |  |  |  |  |  |  |  |  |  |
| INT T1 - INT/ EXT/ ATT^a^ T2 | .35 | .04 | <.001 | [.29, .41] | .03 | .03 | .242 | [- .01, .08] | -.01 | .02 | .794 | [-.04, .03] |
| INT T2 - INT/ EXT/ ATT^a^ T3 | .28 | .05 | <.001 | [.20, .37] | -.05 | .03 | .093 | [-.10, .00] | -.01 | .02 | .793 | [-.04, .03] |
| INT T3 - INT/ EXT/ ATT^a^ T4 | .07 | .10 | .520 | [-.10, .24] | -.01 | .04 | .783 | [-.07, .05] | .00 | .02 | .793 | [-.03, .02] |
| INT T4 - INT/ EXT/ ATT^a^ T5 | .13 | .06 | .034 | [.03, .23] | .02 | .04 | .573 | [-.04, .09] | .00 | .01 | .793 | [-.03, .02] |
| INT T5 - INT/ EXT/ ATT^a^ T6 | .36 | .06 | <.001 | [.26, .46] | .10 | .05 | .025 | [.03, .17] | .00 | .02 | .794 | [-.03, .02] |
|  |  |  |  |  |  |  |  |  |  |  |  |  |
| EXT T1 - INT^a^/ EXT/ ATT^a^ T2 | -.04 | .03 | .199 | [-.08, .01] | .30 | .03 | .000 | [.25, .35] | .14 | .03 | .000 | [.09, .19] |
| EXT T2 - INT^a^/ EXT/ ATT^a^ T3 | -.04 | .03 | .198 | [-.09, .01] | .27 | .05 | <.001 | [.19, .35] | .04 | .04 | .396 | [-.03, .10] |
| EXT T3 - INT^a^/ EXT/ ATT^a^ T4 | -.04 | .03 | .207 | [-.09, .01] | .11 | .09 | .210 | [.03, .25] | .06 | .06 | .322 | [-.04, .16] |
| EXT T4 - INT^a^/ EXT/ ATT^a^ T5 | -.02 | .02 | .198 | [-.05, .01] | .05 | .08 | .553 | [-.09, .18] | -.04 | .06 | .491 | [-.13, .05] |
| EXT T5 - INT^a^/ EXT/ ATT^a^ T6 | -.02 | .01 | .203 | [-.04, .00] | .08 | .09 | .374 | [-.07, .23] | .05 | .04 | .230 | [-.02, .11] |
|  |  |  |  |  |  |  |  |  |  |  |  |  |
| ATT T1 - INT/ EXT/ ATT T2 | .08 | .03 | .011 | [.03, .13] | .04 | .03 | .209 | [-.01, .08] | .23 | .03 | .008 | [.18, .28] |
| ATT T2 - INT/ EXT/ ATT T3 | .01 | .04 | .827 | [-.06, .07] | .11 | .04 | .002 | [.05, .17] | .23 | .06 | <.001 | [.14, .32] |
| ATT T3 - INT/ EXT/ ATT T4 | -.14 | .07 | .062 | [-.26, -.02] | -.05 | .06 | .435 | [-.14, .05] | -.07 | .12 | .557 | [-.27, .13] |
| ATT T4 - INT/ EXT/ ATT T5 | -.10 | .08 | .207 | [-.22, .03] | -.08 | .06 | .207 | [-.19, .02] | .07 | .10 | .503 | [-.10, .24] |
| ATT T5 - INT/ EXT/ ATT T6 | .02 | .05 | .761 | [-.07, .10] | .00 | .06 | .995 | [-.09, .09] | .29 | .06 | <.001 | [.19, .40] |

*Note.* INT = internalizing problems, EXT = externalizing problems, ATT = attention problems. aPaths constrained to be equal for males and females; bPaths constrained to be equal over time.

**Table S11B.** Results from the final constrained RI-CLPM with self-reported problems for females controlling for SES, mental healthcare, and medication use

|  | INT | | | | EXT | | | | ATT | | | |
| --- | --- | --- | --- | --- | --- | --- | --- | --- | --- | --- | --- | --- |
|  | *β* | *SE* | *p* | [95% CI] | *β* | *SE* | *p* | [95% CI] | *β* | *SE* | *p* | [95% CI] |
| *Between-person correlations* |  |  |  |  |  |  |  |  |  |  |  |  |
| INT - EXT/ ATT |  |  |  |  | .56 | .04 | <.001 | [.51, .62] | .61 | .03 | <.001 | [.55, .66] |
| EXT - ATT |  |  |  |  |  |  |  |  | .67 | .03 | <.001 | [.62, .73] |
| *Within-wave (residual) correlations* |  |  |  |  |  |  |  |  |  |  |  |  |
| INT - EXT/ ATT T1 |  |  |  |  | .52 | .03 | <.001 | [.48, .57] | .52 | .03 | <.001 | [.48, .57] |
| EXT - ATT T1 |  |  |  |  |  |  |  |  | .51 | .02 | <.001 | [.47, .55] |
| INT - EXT/ ATT T2 |  |  |  |  | .38 | .03 | <.001 | [.33, .44] | .48 | .03 | <.001 | [.43, .52] |
| EXT - ATT T2 |  |  |  |  |  |  |  |  | .48 | .03 | <.001 | [.44, .53] |
| INT - EXT/ ATT T3 |  |  |  |  | .34 | .04 | <.001 | [.27, .41] | .37 | .05 | <.001 | [.30, .45] |
| EXT - ATT T3 |  |  |  |  |  |  |  |  | .48 | .03 | <.001 | [.43, .54] |
| INT - EXT/ ATT T4 |  |  |  |  | .43 | .05 | <.001 | [.35, .52] | .44 | .05 | <.001 | [.36, .53] |
| EXT - ATT T4 |  |  |  |  |  |  |  |  | .39 | .07 | <.001 | [.28, .50] |
| INT - EXT/ ATT T5 |  |  |  |  | .54 | .04 | <.001 | [.47, .61] | .45 | .04 | <.001 | [.38, .52] |
| EXT - ATT T5 |  |  |  |  |  |  |  |  | .40 | .06 | <.001 | [.30, .49] |
| INT - EXT/ ATT T6 |  |  |  |  | .58 | .03 | <.001 | [.53, .65] | .49 | .03 | <.001 | [.44, .55] |
| EXT - ATT T6 |  |  |  |  |  |  |  |  | .45 | .03 | <.001 | [.40, .51] |
| *Lagged paths* |  |  |  |  |  |  |  |  |  |  |  |  |
| INT T1 - INT/ EXT/ ATT^a^ T2 | .29 | .03 | <.001 | [.24, .34] | .04 | .03 | .242 | [-.02 .09] | -.01 | .02 | .793 | [-.04, .03] |
| INT T2 - INT/ EXT/ ATT^a^ T3 | .27 | .05 | <.001 | [.19, .34] | -.06 | .04 | .095 | [-.12, .00] | -.01 | .02 | .793 | [-.04, .03] |
| INT T3 - INT/ EXT/ ATT^a^ T4 | .06 | .10 | .527 | [-.10, .22] | -.01 | .05 | .834 | [-.09, .07] | -.01 | .02 | .793 | [-.04, .03] |
| INT T4 - INT/ EXT/ ATT^a^ T5 | .15 | .07 | .029 | [.04, .27] | .03 | .06 | .565 | [-.06, .13] | -.01 | .02 | .794 | [-.04, .02] |
| INT T5 - INT/ EXT/ ATT^a^ T6 | .34 | .05 | <.001 | [.26, .43] | .13 | .06 | .021 | [.04, .22] | -.01 | .02 | .794 | [-.04, .02] |
|  |  |  |  |  |  |  |  |  |  |  |  |  |
| EXT T1 - INT^a^/ EXT/ ATT^a^ T2 | -.02 | .02 | .199 | [-.05, .01] | .26 | .03 | <.001 | [.22, .31] | .11 | .03 | <.001 | [.07, .15] |
| EXT T2 - INT^a^/ EXT/ ATT^a^ T3 | -.03 | .02 | .195 | [-.06, .01] | .26 | .05 | <.001 | [.18, .34] | .03 | .04 | .398 | [-.03, .09] |
| EXT T3 - INT^a^/ EXT/ ATT^a^ T4 | -.03 | .02 | .203 | [-.06, .01] | .11 | .09 | .216 | [-.04, .25] | .06 | .06 | .329 | [-.04, .13] |
| EXT T4 - INT^a^/ EXT/ ATT^a^ T5 | -.02 | .01 | .196 | [-.04, .00] | .05 | .08 | .547 | [-.09, .18] | -.04 | .05 | .493 | [-.12, .04] |
| EXT T5 - INT^a^/ EXT/ ATT^a^ T6 | -.01 | .01 | .204 | [-.03, .00] | .08 | .09 | .376 | [-.07, .23] | .04 | .04 | .240 | [-.02, .11] |
|  |  |  |  |  |  |  |  |  |  |  |  |  |
| ATT T1 - INT/ EXT/ ATT T2 | .07 | .03 | .011 | [.02, .11] | .04 | .03 | .207 | [-.01, .09] | .22 | .03 | .008 | [.17, .27] |
| ATT T2 - INT/ EXT/ ATT T3 | .01 | .03 | .827 | [-.04, .06] | .12 | .04 | .002 | [.05, .18] | .22 | .06 | <.001 | [.13, .31] |
| ATT T3 - INT/ EXT/ ATT T4 | -.10 | .05 | .061 | [-.19, -.01] | -.05 | .07 | .442 | [-.16, .06] | -.07 | .13 | .560 | [-.27, .13] |
| ATT T4 - INT/ EXT/ ATT T5 | -.08 | .07 | .211 | [-.19, .03] | -.09 | .07 | .223 | [-.21, .03] | .07 | .10 | .499 | [-.10, .24] |
| ATT T5 - INT/ EXT/ ATT T6 | .01 | .04 | .761 | [-.06, .09] | .00 | .06 | .995 | [-.10, .10] | .30 | .07 | <.001 | [.19, .41] |

*Note.* INT = internalizing problems, EXT = externalizing problems, ATT = attention problems. aPaths constrained to be equal for males and females; bPaths constrained to be equal over time.

1. **Parent-reported problems**

**Procedure**

Parent-reported psychopathology measured at T1 - T3 by the Child Behaviour Checklist ([CBCL]; Achenbach & Rescorla, 2001) was included to investigate potential informant differences in supplemental analyses. The CBCL includes 113 items scored on a 3-point scale (0 = not true; 1 = somewhat/ sometimes true; 2 = very/ often true). Only items that were consistent with the YSR/ASR item selection were included for the calculation of the mean scale scores (cronbach’s alpha’s: internalizing problems α_T1-T3_ = .84 - .86; externalizing problems α_T1-T3_ = .87; attention problems α_T1-T3_ = .74 - .76).

Possible informant differences were investigated by running separate RI-CLPM models with the parent-reported mean problem scores from T1 to T3 following the same approach as the main analyses using self-reports. In short, we first checked whether there was sufficient within-person variance using intraclass correlations (ICCs). Next, RI-CLPMs were run in which the mean problem scores were regressed on their own latent factor with each loading constrained to 1 for each wave (9 within-person latent factors in total). Autoregressive paths, cross-lagged paths, T1 correlation and correlated changes between the latent factors were included as well as three overarching random intercept factors to capture the stable between person differences in internalizing, externalizing, and attention problems. The variances of the observed scores were constrained to zero, thereby capturing all variation in the observed measures by the within- and between-person latent factor structure.

To estimate the stability of path estimates over time, we compared a fully unconstrained model to models in which autoregressive paths, within-wave associations, and/or cross-lagged paths were constrained to be equal over time in a step-wise manner. Differences in model fit were assessed based on the Satorra-Bentler scaled Chi-square difference tests (Satorra & Bentler, 2001), as well as changes in the CFI (ΔCFI ≥.010), RMSEA (ΔRMSEA ≥.015), and SRMR (ΔSRMR ≥.030) following Chen (2007). Model fit was considered good when the model achieved >.90 on CFI, and <.06 on RMSEA, and <.08 on SRMR (Hu & Bentler, 1999). All analyses were run in Mplus 7.31 (Muthén & Muthén, 2015) using robust maximum likelihood (MLR). Missing data was handled with full information maximum likelihood (FIML). Finally, sensitivity analyses were performed while controlling for socioeconomic status, mental healthcare, and medication use following the same approach described for the main analyses.

**Results and conclusions**

The best-fitting RI-CLPM for parent-reported problems (T1 - T3) included stability constraints for all problem scores, in addition to constraints for within-wave associations of internalizing and attention problems, and for all cross-lagged paths except from externalizing to internalizing problems (Table S12, model 13: χ^2^ (17) = 31.389; RMSEA = .020; CFI = .998; SRMR = .018). The results are shown in Figure S1 and supplemental Table S12. In contrast to the RI-CLPM with self-reported problems, homotypic continuity was only significant for attention problems and externalizing problems T1 – T3. Within-wave and between-person associations were comparable to the self-reported results, showing moderate to strong associations. There was, however, no clear decrease in concurrent co-occurrence, as all correlated residuals were constrained to be equal across the waves. Only one cross-path was significant, from externalizing T2 to internalizing T3, indicating little heterotypic continuity. Sensitivity analyses with socioeconomic statis, mental healthcare and medication use resulted in the exact same results, see Table S13.

**Table S12.** Model fit comparisons for RI-CLPMs with parent-reported problems

| Model | | χ^2^ | *df* | RMSEA | CFI | TLI | SRMR | Model comparison | ΔSBχ^2^,  *p*-value | ΔRMSEA | ΔCFI | ΔSRMR | Decision |
| --- | --- | --- | --- | --- | --- | --- | --- | --- | --- | --- | --- | --- | --- |
| 1 | Unconstrained model | 12.503 | *3* | 0.038 | 1 | 0.98 | 0.009 |  |  |  |  |  |  |
| 2 | Stability INT constrained | 15.107 | 4 | .036 | .998 | .985 | .011 | 2 vs 1 | 2.66, p=.103 | .036 | .998 | .985 | Accept |
| 3 | Model 2 + stability EXT constrained | 16.678 | 5 | .033 | .998 | .987 | .012 | 3 vs 2 | 2.48, p=.115 | -.003 | .000 | .002 | Accept |
| 4 | Model 3 + stability ATT constrained | 17.055 | 6 | .029 | .998 | .990 | .012 | 4 vs 3 | 0.52, p=.469 | -.004 | .000 | .003 | Accept |
| 5 | Model 4 + within-wave INT - EXT constrained | 23.269 | 9 | .027 | .998 | .991 | .018 | 5 vs 4 | 6.80, p=.078 | -.002 | .000 | .001 | Accept |
| 6 | Model 5 + within-wave INT - ATT constrained | 26.898 | 11 | .026 | .998 | .992 | .017 | 6 vs 5 | 3.10, p=.213 | -.001 | .000 | .001 | Accept |
| 7 | Model 6 + within-wave EXT - ATT constrained | 28.680 | 12 | .025 | .997 | .992 | .017 | 7 vs 6 | 1.71, p=.191 | -.001 | -.001 | .000 | Accept |
| 8 | Model 7 + cross-lagged INT_t_ - EXT_t+1_ constrained | 29.241 | 13 | .024 | .998 | .993 | .018 | 8 vs 7 | 0.39, p=.534 | -.001 | .001 | .001 | Accept |
| 9 | Model 8 + cross-lagged INT_t_ - ATT_t+1_ constrained | 29.917 | 14 | .023 | .998 | .994 | .018 | 9 vs 8 | 0.00, p=.944 | -.001 | .000 | .001 | Accept |
| 10 | Model 9 + cross-lagged EXT_t_ - INT_t+1_ constrained | 37.659 | 15 | .026 | .997 | .992 | .020 | 10 vs 9 | 11.34, p=.001 | .003 | -.001 | -.002 | Reject |
| 11 | Model 9 + cross-lagged EXT_t_ - ATT_t+1_ constrained | 30.666 | 15 | .022 | .998 | .994 | .018 | 11 vs 9 | 1.05, p=.305 | -.001 | .000 | .000 | Accept |
| 12 | Model 11 + cross-lagged ATT_t_ - INT_t+1_ constrained | 31.116 | 16 | .021 | .998 | .995 | .018 | 12 vs 11 | 0.11, p=.743 | -.001 | .000 | .001 | Accept |
| 13 | Model 12 + cross-lagged ATT_t_ - EXT_t+1_ constrained | 31.389 | 17 | .020 | .998 | .995 | .018 | 13 vs 12 | 0.12, p=.731 | -.001 | .000 | .000 | Accept |

*Note.* INT = internalizing problems, EXT = externalizing problems, ATT = attention problems.

**Table S13.** Results from final constrained RI-CLPM with parent-reported problems (model 13)

|  | INT | | |  | | EXT | | | |  | ATT | | | |  |
| --- | --- | --- | --- | --- | --- | --- | --- | --- | --- | --- | --- | --- | --- | --- | --- |
|  | *β* | *SE* | *p* | | [95% CI] | *β* | *SE* | *p* | [95% CI] | | *β* | *SE* | *p* | [95% CI] | |
| *Between-person correlations* |  |  |  | |  |  |  |  |  | |  |  |  |  | |
| INT - EXT/ ATT |  |  |  | |  | .56 | .03 | <.001 | [.51, .62] | | .50 | .04 | <.001 | [.44, .55] | |
| EXT - ATT |  |  |  | |  |  |  |  |  | | .78 | .03 | <.001 | [.74, .82] | |
| *Within-wave correlations* |  |  |  | |  |  |  |  |  | |  |  |  |  | |
| INT - EXT/ ATT T1 |  |  |  | |  | .46 | .04 | <.001 | [.40, .52] | | .34 | .03 | <.001 | [.27, 40] | |
| EXT - ATT T1 |  |  |  | |  |  |  |  |  | | .49 | .04 | <.001 | [.43, .56] | |
| INT - EXT^a^/ ATT^a^ T2 |  |  |  | |  | .55 | .03 | <.001 | [.50, .60] | | .43 | .03 | <.001 | [.38, .49] | |
| EXT^a^ - ATT^a^ T2 |  |  |  | |  |  |  |  |  | | .50 | .03 | <.001 | [.45, .55] | |
| INT - EXT^a^/ ATT^a^ T3 |  |  |  | |  | .55 | .03 | <.001 | [.50, .60] | | .42 | .03 | <.001 | [.38, .49] | |
| EXT^a^ - ATT^a^ T3 |  |  |  | |  |  |  |  |  | | .50 | .03 | <.001 | [.45, .55] | |
| *Lagged paths* |  |  |  | |  |  |  |  |  | |  |  |  |  | |
| INT T1 - INT^a^/ EXT^a^/ ATT^a^ T2 | .10 | .06 | .062 | | [.01, .19] | .06 | .04 | .148 | [-.05, .13] | | .03 | .04 | .543 | [-.05, .10] | |
| INT T2 - INT^a^/ EXT^a^/ ATT^a^ T3 | .09 | .05 | .066 | | [.01, .18] | .03 | .04 | .543 | [-.04, .11] | | .03 | .06 | .318 | [-.04, .09] | |
|  |  |  |  | |  |  |  |  |  | |  |  |  |  | |
| EXT T1 - INT/ EXT^a^/ ATT^a^ T2 | .04 | .06 | .472 | | [-.06, .15] | .25 | .08 | .001 | [.12, .37] | | .07 | .07 | .317 | [-.04, .17] | |
| EXT T2 - INT/ EXT^a^/ ATT^a^ T3 | .18 | .07 | .006 | | [.07, .29] | .21 | .07 | .001 | [.10, .32] | | .06 | .06 | .318 | [-.04, .15] | |
|  |  |  |  | |  |  |  |  |  | |  |  |  |  | |
| ATT T1 - INT^a^/ EXT^a^/ ATT^a^ T2 | .04 | .04 | .383 | | [-.04, .12] | .00 | .04 | .986 | [-.08, .08] | | .18 | .06 | .002 | [.09, .28] | |
| ATT T2 - INT^a^/ EXT^a^/ ATT^a^ T3 | .04 | .04 | .386 | | [-.03, .10] | .00 | .04 | .986 | [-.07, .07] | | .16 | .05 | .003 | [.07, .25] | |

*Note.* INT = internalizing problems, EXT = externalizing problems, ATT = attention problems. ^a^Paths constrained to be equal over time.

**Figure S1.** Standardized path estimates (standard errors) from RI-CLPM 13 for parent-reported internalizing (INT), externalizing (EXT) and attention problems (ATT). ^a^Paths constrained to be equal over time. All paths significant at *p* <.05.

References

Achenbach, T.M., & Rescorla, L.A. (2001). Manual for the ASEBA school-age forms & profiles. Burlington: University of Vermont.

Achenbach, T.M., & Rescorla, L.A. (2003). Manual for the ASEBA adult forms & profiles. Burlington: University of Vermont.

Chen, F.F. (2007). Sensitivity of Goodness of Fit Indexes to Lack of Measurement Invariance. *Structural Equation Modeling: A Multidisciplinary Journal*, *14*(3), 464–504.

Hamaker, E. (November 2018). How to run a multiple indicator RI-CLPM with Mplus. Retrieved from http://www.statmodel.com/download/RI-CLPM.pdf

Hau, K., & Marsh, H.W. (2004). The use of item parcels in structural equation modelling: Non‐normal data and small sample sizes. *British Journal of Mathematical and Statistical Psychology*, *57*(2), 327–351.

Hu, L., & Bentler, P.M. (1999). Cutoff criteria for fit indexes in covariance structure analysis: Conventional criteria versus new alternatives. *Structural Equation Modeling: A Multidisciplinary Journal*, *6*(1), 1–55.

Muthén, L., & Muthén, B. (2015). *Mplus User’s Guide* (7th Edn.). Los Angeles, CA: Muthén & Muthén.

Rescorla, L., & Achenbach, T. (2004). The Achenbach System of Empirically Based Assessment (ASEBA) for Ages 18 to 90 Years.

Satorra, A., & Bentler, P.M. (2001). A scaled difference chi-square test statistic for moment structure analysis. *Psychometrika*, *66*(4), 507–514.
